# Clinical Application of Diagnostic Imaging of Chiari-Like Malformation and Syringomyelia

**DOI:** 10.3389/fvets.2018.00280

**Published:** 2018-11-28

**Authors:** Clare Rusbridge, Felicity Stringer, Susan P. Knowler

**Affiliations:** ^1^Fitzpatrick Referrals, Godalming, United Kingdom; ^2^School of Veterinary Medicine, Faculty of Health & Medical Sciences, University of Surrey, Guildford, United Kingdom

**Keywords:** complex craniosynostosis syndrome, basilar invagination, COMS, Chiari type I malformation, cine MRI, balanced steady-state free precession sequence, fluid signal-void sign, MRI protocol

## Abstract

Chiari-like malformation (CM) and syringomyelia (SM) is a frequent diagnosis in predisposed brachycephalic toy breeds since increased availability of MRI. However, the relevance of that MRI diagnosis has been questioned as CM, defined as identification of a cerebellar herniation, is ubiquitous in some breeds and SM can be asymptomatic. This article reviews the current knowledge of neuroanatomical changes in symptomatic CM and SM and diagnostic imaging modalities used for the clinical diagnosis of CM-pain or myelopathy related to SM. Although often compared to Chiari type I malformation in humans, canine CM-pain and SM is more comparable to complex craniosynostosis syndromes (i.e., premature fusion of multiple skull sutures) characterized by a short skull (cranial) base, rostrotentorial crowding with rostral forebrain flattening, small, and ventrally orientated olfactory bulbs, displacement of the neural tissue to give increased height of the cranium and further reduction of the functional caudotentorial space with hindbrain herniation. MRI may further reveal changes suggesting raised intracranial pressure such as loss of sulci definition in conjunction with ventriculomegaly. In addition to these brachycephalic changes, dogs with SM are more likely to have craniocervical junction abnormalities including rostral displacement of the axis and atlas with increased odontoid angulation causing craniospinal junction deformation and medulla oblongata elevation. Symptomatic SM is diagnosed on the basis of signs of myelopathy and presence of a large syrinx that is consistent with the neuro-localization. The imaging protocol should establish the longitudinal and transverse extent of the spinal cord involvement by the syrinx. Phantom scratching and cervicotorticollis are associated with large mid-cervical syringes that extend to the superficial dorsal horn. If the cause of CSF channel disruption and syringomyelia is not revealed by anatomical MRI then other imaging modalities may be appropriate with radiography or CT for any associated vertebral abnormalities.

## Introduction

Chiari-like malformation (CM) is a complex skull and craniocervical junction disorder associated with brachycephaly with skull base shortening, low volume caudal fossa and rostrotentorial, caudotentorial and craniospinal crowding. For a detailed written and visual description of the morphogenesis see the review by Knowler et al. (1). The condition has been marred in controversy since the first description, not least by what to call it (2). The eponymic term refers to the first detailed pathological description by Hans Chiari of an analogous human condition (3). The veterinary label, chosen in a round table discussion (2, 4) was considered less restrictive than an anatomical description (for example hindbrain herniation or occipital hypoplasia), which may have proved simplistic or inaccurate in the future. This prediction was true, and over the last two decades our understanding of the complex morphology has deepened and most realize that this condition is more than a cerebellar foramen magnum herniation. As a MRI description, CM should be considered an umbrella term, as the bony and parenchymal changes between and within individuals in each breed are different but have a common tendency toward pain associated with CM and the development of syringomyelia (SM). As such, and with the common feature of being associated with brachycephaly, it was recently proposed that the disorder might be better described as a brachycephalic obstructive cerebrospinal (CSF) channel syndrome (BOCCS) with similarities to brachycephalic obstructive airway syndrome (BOAS) (1).

The analogous disease in humans was considered to be Chiari type I malformation, defined traditionally as a MRI finding of caudal displacement of the cerebellar tonsils inferior to the plane of the foramen magnum by at least 3 mm. However, like the canine disease, this description is problematic especially as there can be symptomatic disease and SM with smaller herniation (termed Chiari type 0). In an attempt to categorize the variations in humans there are now seven recognized types: 0, 1, 1.5, 2, 3, 3.5, and 4 (3–6). However, the distinction between types is challenging especially when the etiology is multifactorial and increasingly there is a call in human medicine that cerebellar tonsil herniation/Chiari malformation should be considered a radiographic sign and the focus of the diagnostic investigation should be to determine the cause of that herniation for example shallow posterior fossa (7), craniosynostosis (8), inherited disorders of connective tissue (9), spinal cord tethering (10), intracranial hypertension (11), or intracranial hypotension (12).

CM is one of the most common causes of SM in the dog which is characterized by the development of cavities in the spinal cord containing a fluid similar to CSF (13, 14) however SM can develop after any obstruction to CSF channels and has been reported in a variety of disorders ranging from acquired cerebellar herniation secondary to intracranial masses (15–18) to spinal arachnoid diverticulum (19, 20) and spinal cord tethering (21). The terminology of SM is equally confused, with some veterinary papers referring to syringohydromyelia or hydrosyringomyelia. These historical terms have been mostly discarded in human medicine (4). Equally confusing is if and when the term hydromyelia is applied. The term *syringomyelia* was first used by Charles-Prosper Ollivier d'Angers (1796–1845) deriving the term from the Greek “*syringe*” meaning tube or pipe, and “*myelio*” referring to the spinal marrow (22, 23). The term *hydroamyelus* was coined by Schüppel in 1865 to describe a dilatation of the central canal (24). In 1875, and after describing spinal cord cavities apparently separate from the central canal and surrounded by gliosis, Simon proposed that hydromyelia be used to describe central canal dilation and distension and that the term syringomyelia be reserved to describe cavities and cystic conditions independent of the central canal (25, 26). In 1876, Leyden concluded that hydromyelia and SM were identical conditions (26, 27) but Kahler and Pick made the observation that a hydromyelia is lined by ependyma whereas glial cells form the wall of SM cavities and recommended keeping a distinction between hydromyelia and SM (26, 28). However, it is difficult to distinguish between hydromyelia and SM, by radiological, clinical, or pathological means and consequently some used the combined terms syringohydromyelia or hydrosyringomyelia, to describe a cavity which is partially lined by ependymal but which also extends into the spinal cord substance (29). Thus, some clinicians argue that the term syringomyelia should only apply to a glia lined cavity separate from the central canal, that hydromyelia be reserved for central canal dilation, still lined by ependyma and that the term syringohydromyelia is correct for a cavity involving a dilated central canal that is partially lined by ependyma. However, post mortem and experimental studies have suggested that the ependyma is disrupted following only minor central canal dilatation and that all syringomyelic cavities are connected to the central canal at some level of the spinal cord (4, 30–32) therefore nowadays the simpler and original term syringomyelia is used by the majority (4). In veterinary medicine a central canal dilatation is defined as a dilatation and distension of the spinal cord with a transverse diameter <2 mm (33).

Identifying a cerebellar herniation and SM on MRI is relatively straightforward and has been defined by the British Veterinary Association and UK Kennel Club with a Health Scheme for breeding dogs based on a grading system for CM, SM, and the maximum transverse diameter of the syrinx if present (33). However, the grading for CM is simplistic and only based on the degree of cerebellar herniation. The diagnosis of symptomatic CM and SM can be challenging in some breeds such as the Cavalier King Charles spaniel (CKCS) because CM, as defined by the BVA/KC Health Scheme is ubiquitous, and SM is prevalent but may be asymptomatic (34, 35). Increasingly it has become apparent that CM alone, like the analogous human condition, can have significant impact on welfare and quality of life (36, 37). Previously we used a MRI morphometric mapping approach to define CM pain and symptomatic SM in the CKCS, Griffon Bruxellois, Chihuahua, and Affenpincher (38–40) and these traits were linked to genomic regions (41, 42). However, translating this research technique to the clinic is challenging as it involves time consuming measurements and there is no objective measure of disease presence/ risk to offspring. Consequently development of a machine learning approach and computer analysis is recommended but development of this will take considerable resources (43, 44). This article serves to review the current knowledge base and provide guidelines to the clinician for the diagnostic imaging of CM-pain and symptomatic SM.

## Current understanding of the morphological changes in canine CM and SM

Many studies, mostly in the CKCS or Griffon Bruxellois, have assessed features of skull and cervical vertebral morphology in relationship to the presence or absence of SM. Not all studies had good control groups, especially the earlier ones. As SM occurs secondary to CM and is more likely in older dogs (34, 35, 45, 46), it is important that the SM-clear cohort consists of dogs MRI scanned when older and typically aged 4 years or more. Equally it is important to accurately phenotype symptomatic dogs which can be challenging as the most common clinical sign is pain which is subjective and in the dog overly reliant on owner reporting (39, 47, 48).

Table 1 summarizes the existing knowledge in relationship to the skull, Table 2 the craniocervical junction and cervical changes, Table 3 the neuro-parenchymal changes and Table 4 the syrinx changes.

**Table 1:** Pathogenesis of Chiari-like malformation and syringomyelia: summary of the existing knowledge base—skull changes.

**Anatomical feature**	**Study finding(s)**	**Possible implication**
Brachycephaly	Brachiocephalic breeds have early closure of the spheno-occipital synchondrosis. In CKCS closure is even earlier (49, 50)	Premature closure of the spheno-occipital synchondrosis will result in a short cranial base (basicranium).
	CKCS have shorter cranium in relation to width compared to other brachycephalic dog breeds (51).	
	Griffon Bruxellois with CM have shortened basicranium and supraoccipital bone, with a compensatory lengthening of the dorsal cranial vault, especially the parietal bone (52)	Basiocranial shortening results in compensatory changes in the rostral cranial fossa which results in a head shape with rostrocaudal doming and is broad in relationship to the length (reduced cephalic index)
	Association between increased cranial height and SM in CKCS, Griffon Bruxellois and Affenpinscher (38, 40)	
	CKCS with broader and shorter skulls and increased rostro-cranial doming are at increased risk of developing SM (53)	
	Association between acute angulation at spheno-occipital synchondrosis (Sphenoid flexure) and SM (40)	May be associated with premature closure of spheno-occipital synchondrosis. This angulation occurs in rodent models where the spheno-occipital synchondrosis is damaged (1, 54)
	Rostral forebrain flattening, short basioccipital bone associated with CM pain	CM pain is associated with increased brachycephaly
	Increased risk of SM with increased proximity of dens to basioccipital bone and/or increased airorhynchy with small more ventrally rotated olfactory bulbs (39)	There are two SM phenotypes: one typified by extreme brachycephalism and one by craniospinal junction deformation
Occipital Crest	Association between reduced occipital crest and SM in CKCS, Affenpinscher and Chihuahua (40)	Suggests insufficiency of the supraocciptal bones and possibly the intraparietal bone
Frontal Sinus	Association between small frontal sinuses and SM in small breed dogs (55)	Suggests that SM may be related to rostrotentorial skull changes rather than being confined to a hind skull abnormality.
Caudal cranial fossa volume	CKCS with CM and SM have a shallower and smaller volume caudal cranial fossa compared to CKCS with CM only and other control breeds (56, 57)	Smaller caudal cranial fossa volume predisposes caudal cranial fossa overcrowding
	CKCS have a strong relationship between hindbrain volume and volume of the rostral part of the caudal cranial fossa and a weak relationship between hindbrain volume and volume of the caudal part of the caudal cranial fossa. In Labrador retrievers and other small breed dogs this relationship is reversed (56, 58)	Small breed dogs and Labrador retrievers compensate for variations in hindbrain volume by modifying growth of the occipital skull. In the CKCS, increased cerebellar size is not accommodated by increased occipital bone development and the tentorium cerebelli compensates by developing / remodeling in a rostral direction
Occipital bone volume	No difference in volume of the occipital bones between CKCS (with and without SM) and French Bulldogs (59)	Does not support theory of occipital bone hypoplasia
Jugular foramina	CKCS with CM and SM have narrowed jugular foramina in comparison with CKCS with CM only (52, 60)	Venous narrowing at the jugular foramina associated with reduced skull base can lead to elevated venous pressure and impaired CSF absorption
Venous sinus volume	CKCS with CM and SM have reduced venous sinus volume in comparison with CKCS with CM only (61)	Reduced venous sinus volume could result in intracranial hypertension and impaired CSF absorption

**Table 2:** Pathogenesis of Chiari-like malformation and syringomyelia: summary of the existing knowledge base—craniocervical junction and cervical changes.

**Anatomical feature**	**Study finding(s)**	**Possible implication**
Proximity of atlas to skull (atlanto-occipital overlapping)	SM risk increases with decreased distance between atlas and occipital bones (39, 62–64)	Reduced distance between the skull and the cervical vertebrae increases risk of SM
	Greater distance between atlas and basioccipital bone is protective against SM (39)	
	CKCS with SM have shorter distance between the spheno-occipital synchondrosis and atlas (40)	
Odontoid peg impingement of ventral subarachnoid space/neural tissue	Commonly seen in association with CM (40, 56, 65)	Contributes to overcrowding and conformation change of craniospinal junction with loss of cisterna magnum
	Greater distance between atlas and odontoid peg is protective against SM (40)	
	Odontoid peg is more acutely angled, contributing to craniospinal disproportion, medullary elevation and cervical flexure (40)	
Proximity of dens to atlas	SM risk increases with decreased distance between odontoid peg and atlas in Affenpincher (40)	
Dorsal impingement subarachnoid space/spinal cord (atlantoaxial bands) at C1-C2	Commonly seen in association with CM and more prominent in extended than flexed positions (56, 62, 63, 65, 66)	Significance undetermined
Width of spinal canal	Increased width of spinal canal at C2-C3 and C3 in CKCS with SM (67)	Questionable clinical significance
Atlantoaxial subluxation	Occasional comorbidity with CM (68)	No significant association with SM
Size of C2 spinous process	Significantly smaller in CKCSs than in non-CKCS breeds (68)	
Angulation at C2-C3	No correlation (67)	

**Table 3:** Pathogenesis of Chiari-like malformation and syringomyelia: summary of the existing knowledge base—neuroparenchymal changes.

**Anatomical feature**	**Study finding(s)**	**Possible implication**
Parenchymal (brain) volume	The absolute and relative volume of the CKCS skull is similar to other brachycephalic toy dog breeds but CKCS have a greater volume of parenchyma within the caudal cranial fossa (69) and CKCS with early onset SM have a larger volume of parenchyma within a smaller caudal cranial fossa compared to older CKCS with CM only (57, 61, 70)	Mismatch in skull and brain volume is associated with development of SM.
Cerebellar volume	CKCS have relatively increased cerebellar volume compared to other control breeds and this is associated with development of SM (71)	Caudal cranial fossa overcrowding is associated with development of SM
Cerebellar herniation	Typically present but size does not predict SM (62, 72, 73)	Obstruction of CSF channels though the foramen magnum contributes to the pathogenesis of SM but there must also be other predisposing factors.
	Positive association with the size of foramen magnum and size of cerebellar herniation (62)	Overcrowding of the caudal cranial fossa causes supraoccipital bone resorption (occipital dysplasia) and widening of the foramen magnum over time.
	The length of the cerebellar herniation increases with time. The size of the foramen magnum also increases (74, 75)	
Cerebellar pulsation	CKCS with CM and SM have significantly greater pulsation of the cerebellum compared to CKCS with CM only and other control breeds (76)	Abnormal cerebellar pulsation could lead to a mismatch in the timing of the arterial and CSF pulse waves predisposing SM (77, 78)
Position of cerebellum relative to occipital lobe	Rostrotententorial craniocerebral disproportion results in the occipital lobes being displaced caudally so that cerebellum is invaginated under the occipital lobes (40).	Overcrowding in both cranial and caudal fossa affects position of cerebellum
Medullary elevation (medullary kinking)	Higher medullary kinking index is associated with clinical signs in CKCS and Chihuahuas (47, 79)	Dogs with higher medullary elevation / kinking are more likely to have clinical signs
Caudal medulla (obex) position	Association between more caudal brainstem positions and presence of SM (79)	Caudal displacement of the obex may increase risk of SM
CSF flow	Higher peak CSF flow velocity at the foramen magnum with a lower CSF flow velocity at C2–C3 predicts SM (80)	SM is associated with alterations in the CSF velocity profile
	Turbulence at the foramen magnum and at the C2–C3 disc significantly associated with SM (80)	
	Presence CSF signal-void sign in mesencephalic aqueduct on T2W is associated with SM and increased ventricular size (81)	
Ventricle dimensions	In CKCS ventricle dimensions are positively correlated with syrinx width (57)	Evidence that SM is related to CSF channel obstruction
	Are not correlated with seizures (nor is caudal cranial fossa overcrowding) (82)	Epilepsy and CM in CKCS should be considered unrelated

**Table 4:** Pathogenesis of Chiari-like malformation and syringomyelia: summary of the existing knowledge base—syrinx features.

**Anatomical feature**	**Study finding(s)**	**Possible implication**
Syrinx presence	If a syrinx is detected in a assymptomatic dog having MRI screening prior to breeding then there is a higher change that this dog may develop clincial signs of CMSM later in life compared to a dog without a visible syrinx (83) However dogs with no clinical signs at the age of 6 are more likely to remain asymptomatic (45)	Early development of syringomyelia is more likely to be associated with clinical signs even if the dogs is initialy assymptomatic
Site of syrinx	In CKCS, SM tends to develop first within the C2–C4, T2-T4, and T12-L2 spinal-cord segments (77, 84, 85).	SM development may be associated with subarachnoid space narrowing and/or change in the angulation of the vertebral canal
	Axial stress increases in the cranial cervical and cervico-thoracic regions where the spinal cord has most curvature (86)	Increased axial stress at the site of spinal curvature may explain the syrinx distribution in the CKCS
	In CKCS 76% of dogs with a syrinx at C1-C4 also had a syrinx in the C5-T1 and T2-L2 regions and 49% had a syrinx in the L3-L7 region (85)	In CKCS MRI imaging of the cranial cervical region only has high sensitivity for detection of SM however the extent of the disease may be underestimated
Syrinx size and symmetry	Pain is positively correlated with SM transverse width and symmetry on the vertical axis (32, 87)	Dogs with a wider asymmetrical SM more likely to experience pain
	Phantom (fictive) scratching is associated with a mid-cervical spinal cord segment syringe with extension to the superficial dorsal horn (88)	Phantom (fictive) scratching is associated with damage to the mid-cervical superficial dorsal horn
	Dogs with a wide syrinx and dorsal gray column damage are also more likely to have cervicothoracic scoliosis (87)	Gray column damage can result in an imbalance of proprioceptive information and cervical dystonia (89)

## Summary of existing knowledge of the morphological changes in canine CM and SM

The key feature of canine CM is craniosynostosis of particularly the spheno-occipital synchondrosis. However, premature closure of the spheno-occipital and intersphenoidal synchondrosis define the canine brachycephalic skull (1, 49) and CM does not occur in all brachycephalic dogs. Therefore, CM is a more complex disorder and likely involves other premature suture closure (especially the lamboid) or other causes of insufficient cranium. The skull insufficiency results in rostrotentorial crowding which further reduces the functional caudotentorial space and causes hindbrain herniation. It is complicated by craniocervical junction deformation including change in angulation of the dens and increased proximity of the atlas to the skull and loss of the cisterna magna. Loss of the cisterna magna or other alteration in the CSF volume will affect the compliance of the CNS (90). In addition some predisposed breeds such as the CKCS have comparatively big brains (69, 71, 91). The pathogenesis of SM associated with CM is undetermined but is predisposed by two phenotypes (or combination); the first by extreme brachycephaly and the second by craniocervical junction deformation (39). This may be influenced by poor venous drainage, intracranial hypertension, changes in CNS compliance and conformational features of the spinal canal. Although CM is considered a naturally occurring model of adult Chiari type 1 malformation it is much closer to the hindbrain herniation seen with complex craniosynostosis such as Crouzon's syndrome (92).

## Diagnostic imaging of CM and SM

### Radiographs

**Figure 1:**
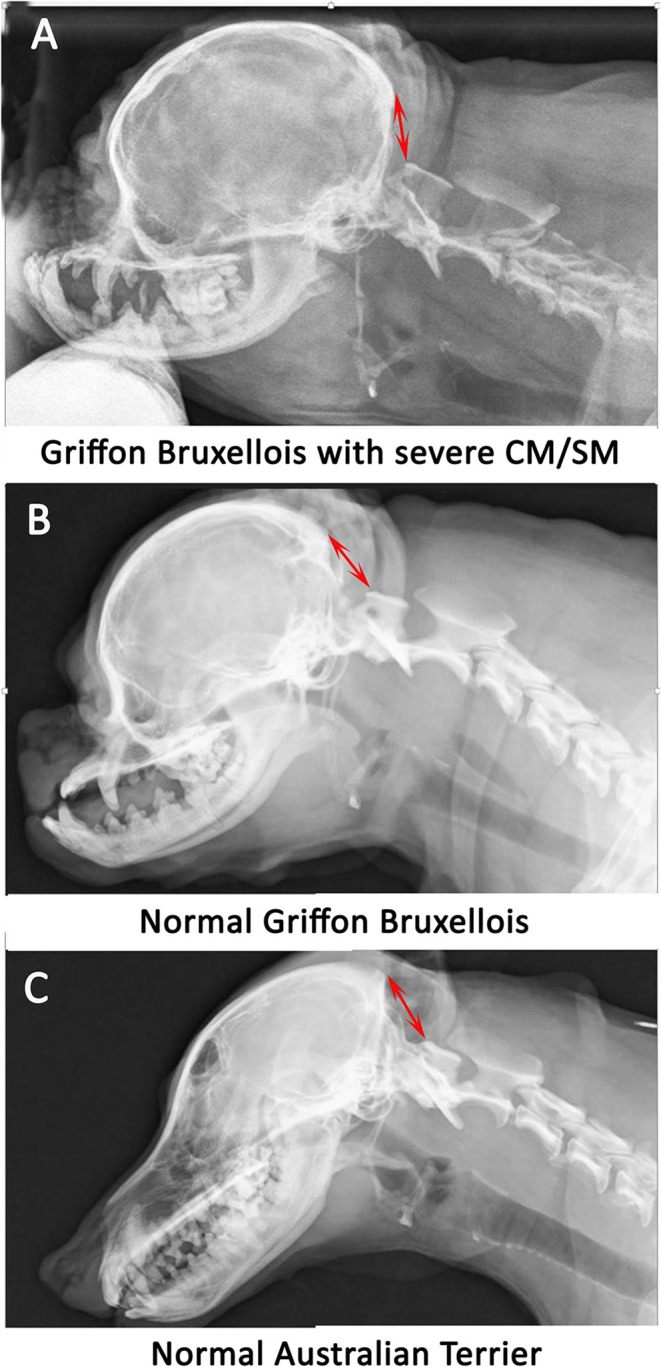
Lateral skull and cranial cervical spinal radiographs in a Griffon Bruxellois with MRI confirmed severe CM and SM **(A)**, a normal Griffon Bruxellois **(B)**, and an Australian terrier **(C)**. The head is in extension as the space between the dorsal atlas and the occiput increases with flexion. A red arrow is placed between the occiput and the dorsal tubercle of the atlas for each dog. The atlas is considerably closer to the skull in the SM affected dog.

**Figure 2:**
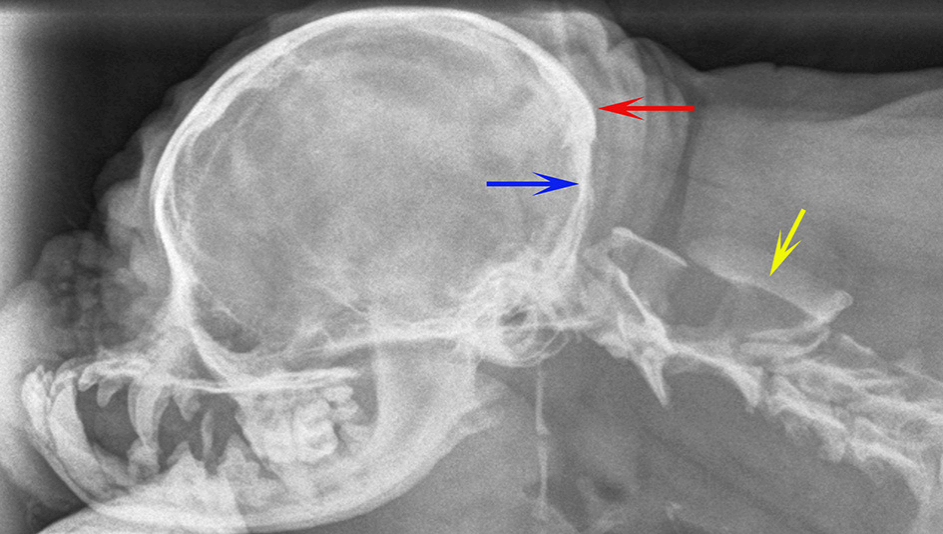
Lateral skull and cranial cervical spinal radiographs of a 6 year old female Griffon Bruxellois presented with tetraparesis and following MRI diagnosed with symptomatic CM and SM. The skull has rostro-cranial doming and a “copper beaten” appearance due to convolutional markings relating to the gyri and presumed raised intracranial pressure. The supraoccipital bone is flattened (blue arrow) and the occipital crest is small (red arrow). A large cervical syrinx has resulting in widening of the cervical spinal canal with thinning and scalloping of the vertebrae (yellow arrow). A MRI confirmed CM, SM, and ventriculomegaly. The maximum width of the syrinx in the cervical spinal cord in a transverse section at the level of C2 was 8 mm.

**Figure 3:**
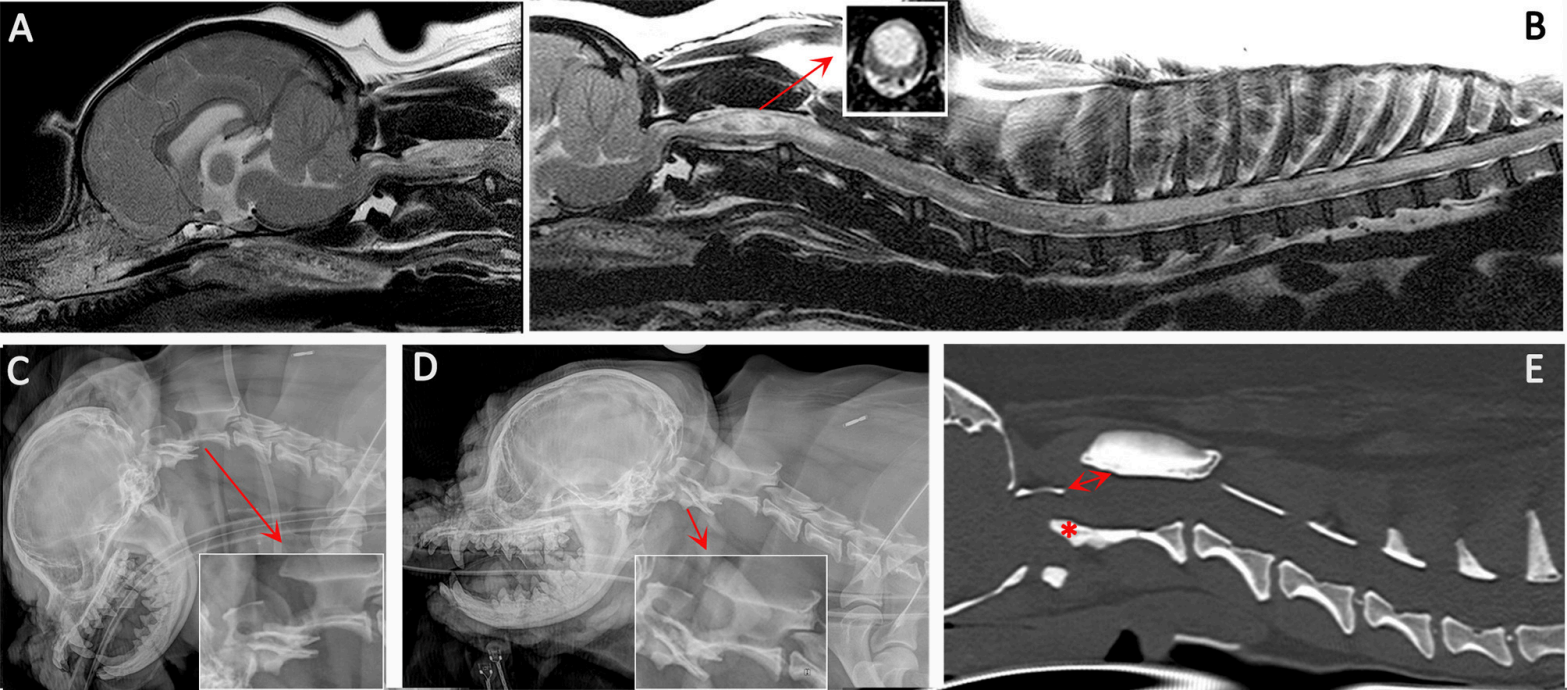
Diagnostic imaging from a 5 year female CKCS presented with lethargy, ataxia, cervicotorticollis, and phantom scratching and following MRI diagnosed with symptomatic CM and SM. **(A)** T2-weighted mid-sagittal MRI of the brain and cranial cervical spinal cord. **(B)** T2- weighted mid sagittal MRI of the hindbrain and spine from C1 to T10. **(C)** Lateral skull and cervical spinal radiograph flexed at the atlantoaxial joint. **(D)** Lateral skull and cervical spinal radiograph extended at the atlantoaxial joint. **(E)** CT reformatted in the sagittal plane of the skull and cervical spine. MRI imaging **(A,B)** confirmed CM and SM with a large mid cervical SM involving the superficial dorsal horn thus explaining the cervicotorticollis and phantom scratching (insert). MRI also suggested atlantoaxial instability with spinal cord compression by the odontoid peg. Atlantoaxial instability was confirmed by a dynamic radiographic study **(C,D)**. The CT was obtained for pre-surgical planning. The dorsal displacement of the odontoid peg (asterisk) and dorsal opening (arrow) between the atlas and axis can be appreciated (Siemens Magnetom Symphony, A Tim System, 1.5 T, Erlangen, Germany; Toshiba Aquilion Prime 160 slice, Otawara, Japan).

Radiographs are not recommended for the investigation of CM and SM. However, if they have been obtained, for example by the general practitioner in the work-up for cervical pain, then there may be features that are suggestive of CM and SM such as flattened supraoccipital bone and close proximity of the atlas to the skull (Figures 1, 2). In the instance of severe SM there may be widening of the cervical spinal canal and remodeling and scalloping of the vertebrae due to increased intraspinal pressure (93) (Figure 2). Dynamic (flexion and extension) atlantoaxial radiographs may be indicted to assess stability of the atlantoaxial joint especially if a foramen magnum decompression is planned (Figure 3).

### Ultrasound

In veterinary medicine ultrasound does not feature in management of CM and SM as it does in human medicine, where 3D ultrasound it is used intraoperatively to tailor a foramen magnum decompression and optimize re-establishment of CSF flow (94, 95). Ultrasonography of the atlanto-occipital junction has been described, proving it is possible to discern the cerebellar herniation (96). Hypothetically, ultrasound guidance could prove a useful aid for placement of a shunt into a syrinx or ventricle via a laminectomy or craniectomy.

### Computed tomography (CT)

CT should be performed if a vertebral malformation is suspected in association with SM especially if implanted surgical fixation is likely and to facilitate planning of this procedure (Figure 3). Most descriptions of CT in the investigation of CM and SM have been to answer a research hypothesis or question (51, 59, 60, 63, 97). Although CT has limited value in assessing CM and SM other than confirming a cerebellar herniation (98) and defining craniocervical junction abnormalities (63), hypothetically it could play a future role in health screening pedigree dogs assuming accurate morphometric analysis/machine learning can be translated from MRI studies and especially if risk of future disease could be predicted (99).

### Myelography

**Figure 4:**
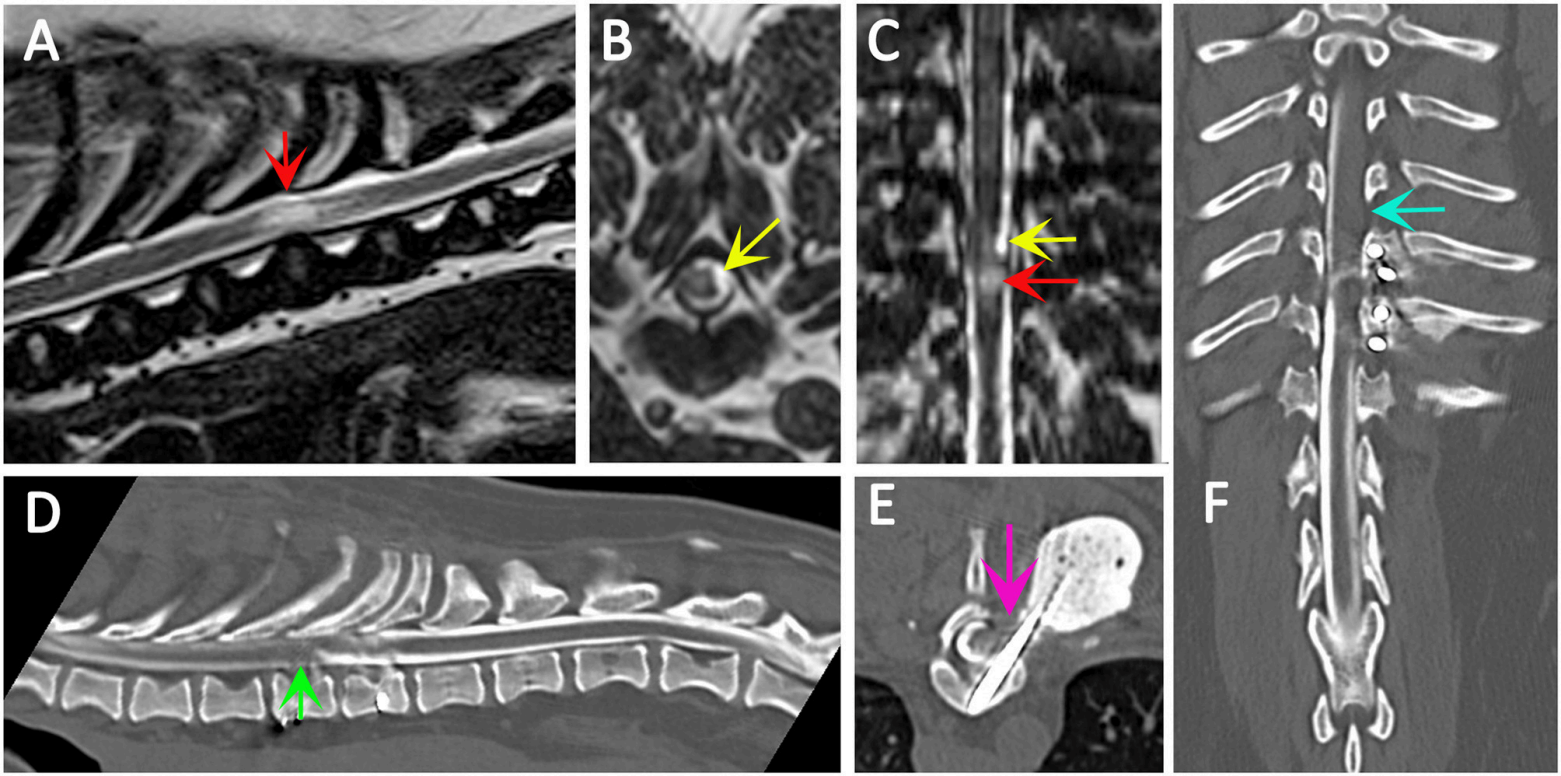
Use of CT myelography to investigate arachnoid adhesions in in a 10 year old female Pug dog presented with signs of a thoracic myelopathy and that had previous surgical management for spinal arachnoid diverticulum and vertebral instability. Six months previous this dog had previously been managed surgically for a spinal arachnoid diverticulum (red arrow) with associated spinal cord edema/presyrinx (yellow arrow) at the level of T9/T10. She had been presented originally with a 2 year progressive history of myelopathy and the arachnoid diverticulum was considered associated with vertebral instability. Surgical management included marsupialization of the arachnoid diverticulum and spinal stabilization with Interface Pins (IMEX Veterinary Inc, Long View Texas) and Simplex-tobramycin bone cement (Howmedica, Limerick, Ireland). After an initial improvement the dog deteriorated and the metal implant precluded repeat MRI. CT myelography was performed from a lumbar injection. There is a reduced flow of contrast cranially (green arrow) and block to flow of the contrast material on the left side (blue arrow) and transverse images suggest adhesions between the spinal cord tissue and laminectomy site confirmed at surgery (pink arrow). **(A)** T2-weighted mid-sagittal MRI of the spinal cord from T6 to L1. There is a focal area of spinal cord edema (presyrinx) at the level of T9/T10 (red arrow). **(B)** Transverse 3D-CISS image at the level of T9/T10. There is a focal dilatation of the subarachnoid space at T9/T10 (yellow arrow). **(C)** Dorsal 3D-CISS image at the level of T7 to T13 demonstrating spinal cord edema (presyrinx) at the level of T9/T10 (red arrow) and suspected arachnoid diverticulum cranial to it (yellow arrow). **(D)** Midsagittal computed tomographic myelography from T6 to L2; there is reduced flow of iodinated contrast material cranial to T9 (green arrow). **(E)** transverse computed tomographic myelopathy at the level of T9. There is an adhesion between the spinal cord and laminectomy site (pink arrow). **(F)** Dorsal computed tomographic myelography from T6 to L2; there is a block of iodinated contrast material cranial to T9 (blue arrow) (Siemens Magnetom Symphony, A Tim System, 1.5 T, Erlangen, Germany; Toshiba Aquilion Prime 160 slice, Otawara, Japan). Image acknowledgment Dr Anna Tauro and Dr Colin Driver, Fitzpatrick Referrals.

Myelography and CT myelography can be used to investigate SM secondary to arachnoid diverticulae and webs/bands (100, 101) and CT myelography is the procedure of choice in the investigation of idiopathic SM if anatomical or cine MRI techniques are not available or have not revealed the cause of the CSF channel obstruction (102, 103). In veterinary medicine the most common application of CT myelography is when MRI is inappropriate because of metal implants and the owner wants to pursue further surgical management. An arachnoid web/band is indicated by a (often dorsal) flow block indicating CSF obstruction in combination with displacement or change in spinal cord caliber (Figure 4). An arachnoid diverticulae is a CSF containing space, lined by arachnoid mater which communicates with the subarachnoid space via a narrow “neck” enlarging via a one-way valve effect. With myelography the diverticulae will fill with contrast (101, 103, 104). Intrathecal contrast medium injections are more challenging when the spinal subarachnoid space is narrowed as a consequence of SM and should be undertaken by an experienced operator. CT myelography is not indicated in the investigation of SM secondary to CM.

### Magnetic resonance imaging (MRI)

MRI is undoubtedly the modality of choice to investigate CM and/or SM. On making a diagnosis of CM/SM there are six aims for the clinician.

**Figure 5:**
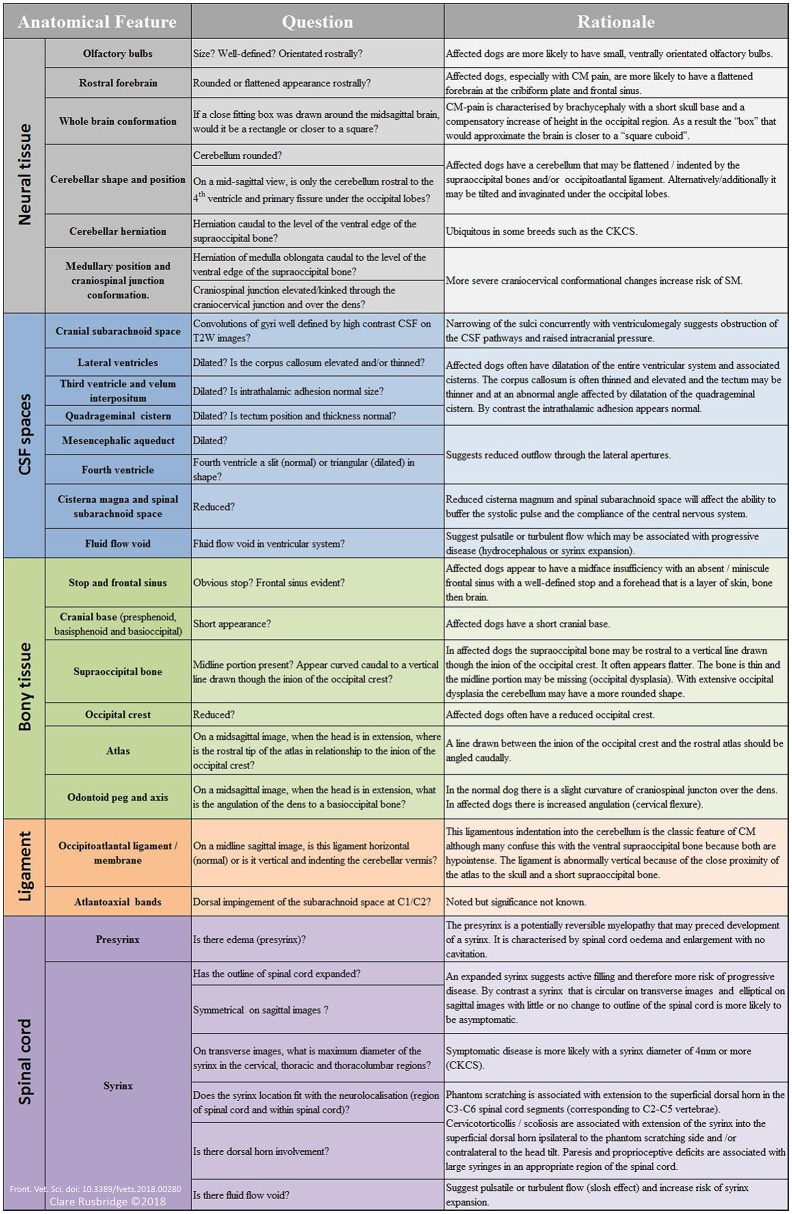
Suggested interpretation of MRI for diagnosis of CM pain and symptomatic SM—the authors' method. With the exception of the basic scoring by the CMSM health scheme (33), there is no objective measure for the diagnosis of CM pain. The diagnosis is made by exclusion and the weight of clinical and MRI evidence. Not all dogs will have all the features. The diagnosis of symptomatic SM is more objective reflecting the size and neuro-localization. For the supporting scientific justification see Tables 1–4 however this method is also based on the authors own observations and interpretations of which the reader should take account.

1. To assess and document the anatomical changes (Figure 5, Supplementary Figure 1).
2. To determine the cause of the SM. SM is an acquired disease which occurs secondary to CSF channel disruption therefore the aim of MRI is to determine the site of that obstruction. Typically disruption at the craniocervical junction due to CM results in syrinx development in the cranial cervical region (85).
3. To determine the full extent of disease, for example the caudal extent of the syrinx in the event of holocord SM (85).
4. Eliminate other potential causes of the clinical presentation and neurological localization, for example intervertebral disc disease as an alternative explanation for spinal pain.
5. Assess whether the radiological findings are consistent with the neurological localization and severity, for example forebrain signs such as seizures or cranial nerve deficits such as facial nerve paralysis cannot be explained by SM which is a spinal cord disease.
6. To determine if other diagnostic modalities are recommended, for example CT to characterize bony abnormalities that might need surgical stabilization and therefore planning.

## MRI protocols to investigate CM and SM

Diagnostic MRI evaluation of CM and SM should include imaging of brain and spinal cord in at least two orientations and include protocols to produce static T1-weighted and T2-weighted sequences (102). Sagittal and transverse imaging of the brain including the craniocervical junction is essential to evaluate the rostrotentorial and caudotentorial overcrowding, CSF spaces and any compromise of the craniospinal junction. Spinal MRI establishes the presence, maximum width on transverse images, dorsal horn involvement and longitudinal extent of the syrinx/presyrinx (Table 5 and Supplementary File 1). It is recommended that at the time of scanning that the microchip or tattoo number (confirmed by the veterinary surgeon) is included on the DICOM images in addition to the Kennel Club registration number if the dog is registered. This is to permit submission to an official CMSM health scheme should the owner request it (33).

**Table 5:** Diagnostic imaging protocols for investigation of CM and SM.

**Area**	**Sequence**	**Assessment of**
**ESSENTIAL PROTOCOL**		
Brain and craniocervical junction	- **TW2 (high field) or T1W (low field) sagittal and transverse** - Maximum slice thickness 4 mm	- Conformation brain and craniocervical junction - CSF spaces - Other differential diagnoses
Cervical vertebral column	- **TW2 and T1W sagittal** - **TW2 (high field) or T1W (low field) transverse** with the block perpendicular to the spinal cord though the maximum width of the syrinx if SM is present, or as a block centred on C3 and extending from at least mid-point of the vertebral body of C2 - Maximum slice thickness 4 mm	- Presence of SM, central canal dilation, spinal cord edema (presyrinx) - Measurement of maximum transverse width SM - Spinal cord dorsal horn involvement by SM - CSF spaces including cisterna magna - Other differential diagnoses
Thoracic vertebral column	- **TW2 (high field) or T1W (low field) sagittal with or without transverse** with the block perpendicular to the spinal cord though the maximum width of the syrinx if SM is present. Maximum slice thickness 4 mm	- Presence of SM or central canal dilation - Measurement of maximum transverse width SM - Spinal cord dorsal horn involvement by SM - Subarachnoid space - Other differential diagnoses
**ADDITIONAL OR ALTERNATIVE SEQUENCES**		
**Brain and craniocervical junction**	- **Three-dimensional, T1-weighted, gradient-echo sequence (MPRAGE)** - **Maximum slice thickness 1mm**.	- Used by the authors as an alternative to T1W spin echo sagittal and transverse
	- **Fluid-attenuated inversion recovery (FLAIR)** - Maximum slice thickness 4 mm	- To assess potential periventricular hyperintensive lesions for example with acute hydrocephalous or inflammatory disease
Lumbar and lumbosacral vertebral column	- **TW2 (high field) or T1W (low field) sagittal with or without transverse** with the block perpendicular to the spinal cord though the maximum width of the syrinx if SM is present. Maximum slice thickness 4 mm	- If neurolocalization suggests and/or SM extends caudally to lumbar spinal cord and to investigate spinal cord tethering by the filum terminale if suspected
Area of suspected mass and /or spinal cord edema of unknown aetiology	- **Paramagnetic contrast**	- Cystic intramedullary tumours - Other differential diagnoses
Vertebral column	- **Half-Fourier acquisition single-shot turbo spin-echo (HASTE)**	- Used to some to make a more rapid assessment of the subarachnoid space
Area of suspected arachnoid web/band/dilatation	- **Balanced steady-state free precession sequences (bSSFP)**	- Recommended (high field)/essential (low field) to assess CSF space if arachnoid web suspected
	- **MRI flow studies (Cine MRI)**	- **Phase contrast cine MRI** - Identify region of flow abnormalities/obstruction - Prognostication
		- **Cine bSSFP** - Define the arachnoid webs or bands
	- **CT myelography**	- If bSSFP and/or Cine MRI has not identified or not possible because of metal implants and if owner/veterinarian wishes to pursue possible surgical management

*T1W—T1-weighted, TW2—T2 weighted. It is recommended that at the time of scanning that the microchip or tattoo number (confirmed by the veterinary surgeon) is included on the DICOM images in addition to the Kennel Club registration number if the dog is registered. This is to permit submission to an official CMSM health scheme should the owner request (33). The parameters for a 1.5 T machine are detailed in Supplementary File 1*.

Protocols will vary between low and high field machines because of the difference in anatomical definition. T2-weighted sequences will be preferred for high field machines and T1-weighted sequences for low field machines. However, at least one region of the spinal cord, typically a sagittal sequence of the cervical region, that includes both T1 and T2-weighting is essential to determine that the signal characteristics of the fluid filled cavity is identical to CSF and to eliminate other causes of hyperintensity on T2-weighted images for example edema associated with meningoencephalomyelitis of unknown origin. As a general rule, measurements of the width of CSF spaces are considered more accurate on T1-weighted images, however T2 weighted images are more sensitive to the presence of excessive fluid within the neural tissue and in particular presyrinx (presyringomyelia) which may eventually form a syrinx (102, 105). Limited “low cost” imaging of CM or SM with a 3-sequence protocol of the hindbrain and cranial cervical spinal cord is offered by some institutions for dog breeders that wish to screen their breeding stock (33, 106), however this minimal protocol does not provide information about the brain or syrinx involvement of the thoracic and lumbar regions and is not recommended for the dog presented to a veterinarian for a diagnostic work of suspected CM or SM. Factors that influence the ability to make an accurate assessment of CM and SM are detailed in Table 6.

**Table 6:** Factors that influence the ability of make an accurate assessment of CM and SM on MRI.

**Type**	**Example**	**Notes**
Protocol	Slice thickness	In the sagittal plane thinner slices (3 mm or less) are preferred to achieve 2–3 sections through the spinal canal and more chance of lesion detection. Thicker slice thickness may miss a small intramedullary lesion in an dog with a spinal cord diameter ranging between 4.1 and 10.3 mm (depending on site imaged and size of animal) (107, 108). However, MRI machines of 1 Tesla or less cannot provide thin slices with sufficient signal-to-noise ratio. Using a balanced steady state feed precession sequence (bSSFP) in addition to a conventional gradient echo and spin echo sequences may help overcome this challenge (109).
	Sequence	Protocols that achieve both T1W and T2W weighting are required to be confident in detecting a fluid filled cavity within the spinal cord and conformational changes with CM.Anatomical imaging of the entire brain is recommended and full extent of the syrinx should be determinedTransverse images perpendicular to the spinal cord though the syrinx are required to assess the transverse width and extent of spinal cord involvement.
Magnetic field strength	Low field vs. high field	Signal-to-noise ratio and spatial resolution is improved when imaging with higher magnetic field-strength which allows shorter imaging times for a given resolution and/or higher resolution for a given imaging time. In addition, higher signal-to-noise ratio allows better resolution with smaller voxel size and thinner slice thickness (110).
Operator factors	Inexperience/lack of training	In veterinary medicine it is possible to operate a MRI service without any Specialist qualification. By contrast an experience MRI technician has undertaken a 3–4 year radiography degree plus additional post-graduate MRI training.
	Diligence	Out with other reasons for decreasing imaging time (economic/duration of anesthesia), operator inclination is a factor for example image quality can be improved by increasing the number of averages (NEX/NSA) which will subsequently increase the acquisition time.
Interpreter factors	Inexperience/lack of training	Failure to recognize significant lesions or over-interpretation of other features for example attributing SM to epilepsy, facial nerve paralysis, fly catching and other brain disorders or interpreting a generalized pruritus as due to SM.In humans it is reported more likely that a cervical syrinx is missed with techniques for whole spine sagittal scanning with focused lumbar spinal MRI where the physician is biased from the history for a lumbar lesion (111). Conversely, asymptomatic localized widening of the central canal may be observed in both humans and dogs (34, 112).
Patient factors	Skull and air interface	May cause susceptibility artifacts, especially on gradient echo sequences.
	Small brain and narrow spinal cord	Slice thickness should be proportional to the brain volume to achieve images with diagnostic quality i.e., animals with smaller brain volume require thinner slices. In a low field MRI this may be challenging and a bSSFP sequence is recommended.
	Positioning	Assessment for CM is normally obtained with the head in extension as reproducibility is easier and anesthesia is safer as the airway may be compromised in the flexed position (33). However, cerebellar herniation and CSF space between the cerebellum and brainstem are significantly increased in the flexed position (113).
	Microchips, orthopedic implants and shrapnel	Ferromagnetic materials cause susceptibility artifacts which may compromise interpretation especially identity microchips for studies of the cervical spine. In low field MRI a T1W turbospin echo sequence is recommended (114) and for high field MRI, spin echo sequences have smaller artifacts than gradient echo sequences (115). Titanium or oxidized zirconium implants have less susceptibly artifact than cobalt-chromium alloy implants (116)
	General anesthetic	Increased time under general anesthesia may increase risk to patient and cost thus limiting length of any MRI protocol.
Motion related artifacts	Neural tissue	Standard MRI sequences are optimized for good spatial and contrast resolution, however this results in blurring of moving structures which compromises the ability to detect fine structures such as arachnoid webs and other adhesions, septations in the syrinx or appreciate dynamic compression (104). It may also blur the edges of a syrinx cavity.
	Intrasyringeal fluid flow void	Pulsatile or turbulent motion of fluid within the syrinx produces low signal on T2W images because of an absence of activated protons in that region (117)
	Intraventricular CSF pulsation artifact	Intraventricular hyperintensity on FLAIR imaging which result in false—negative/positive interpretations of ventricular pathology and is a particular problem for FLAIR performed on low field MRI (118). The most common cause is pulsatile movement and un-inverted CSF flowing into the slice between the pulses (119). It can also occur because of inadequate inversion of CSF magnetization at the edge of the transmitted coil or because of increased CSF protein or oxygen (breathing 100% oxygen) which shortens T1 (119).

T2 Fluid-attenuated inversion recovery (FLAIR) imaging of the brain is a sequence which uses an inversion pulse and long echo time to suppress normal CSF signal on a heavily T2-weighted image. Pathology is suggested by high signal against background of normal signal from the brain and low or zero signal from the CSF (119). A FLAIR is not an essential part of CMSM protocol but is indicated in the assessment of acute hydrocephalus to demonstrate periventricular interstitial edema and to aid identification of any causative or associated lesion (120). FLAIR sequences are also indicated if meningoencephalomyelitis is suspected (121).

Paramagnetic contrast enhancement may be indicated especially if (i) there is evidence of a mass; (ii) if the cause of the CSF channel obstruction is not apparent; (iii) there is a presyrinx and need to eliminate other causes of spinal cord edema. Spinal intramedullary tumors may be cystic and it is important to distinguish these from SM (122, 123).

**Figure 6:**
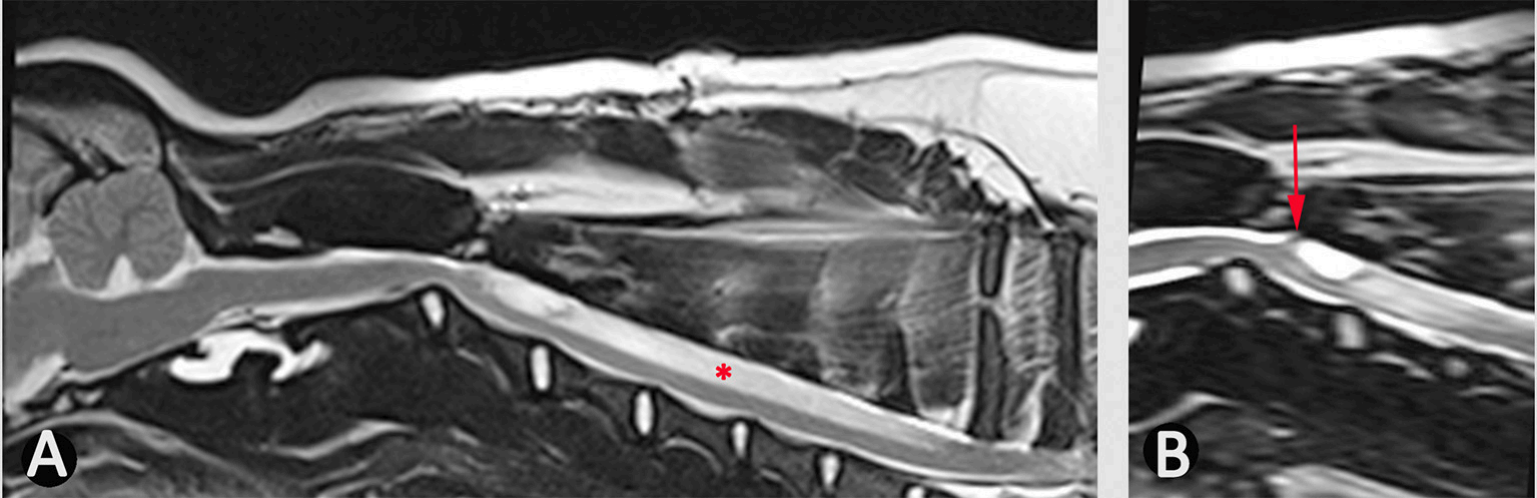
Use of bSSFP (3D CISS) to identify a spinal cord adhesion in an 18 month old male Pug dog presented with signs of cervical myelopathy. This dog had previously been managed surgically for a spinal arachnoid diverticulum at the level of C2/C3 (age 5 months). **(A)** T2-weighted mid-sagittal MRI of the hind brain and cervical spinal cord **(B)** mid-sagittal 3D-CISS image at the level of C2–C4. There is a syrinx between C3 and C7 (asterisk). The 3D-CISS image highlights the likely cause of this syrinx as an adhesion between the spinal cord and laminectomy site (arrow) (Siemens Magnetom Symphony, A Tim System, 1.5 T, Erlangen, Germany).

If the cause of CSF channel disruption is not apparent, then balanced steady-state free precession sequences (bSSFP) such as FIESTA (Fast Imaging Employing Steady-state Acquisition) or 3D-CISS (Three-Dimensional Constructive Interference in steady state) should be employed to improve detection of arachnoid webs and diverticulae (109, 124). These are a three dimensional gradient echo sequence that produces high contrast between the CSF and structures within the subarachnoid space. They have less flow void artifact associated with turbulent CSF and allow higher detection rates of arachnoid webs and other adhesions (102, 125) (Figure 6) and for low field MRI where obtaining good signal-to-noise and spatial resolution is a challenge, it is recommended that a bSSFP sequence be included in any protocol that evaluates the CSF channels (or disruption of) (109). Half-Fourier acquisition single-shot turbo spin-echo (HASTE) sequences are heavily T2W sequences that produced a myelographic effect and can be obtained in a very short time. They are also described as being useful to detect arachnoid diverticulae (126). However, the trade-off for such short imaging times is lower spatial resolution and T2 blurring artifact.

**Figure 7:**
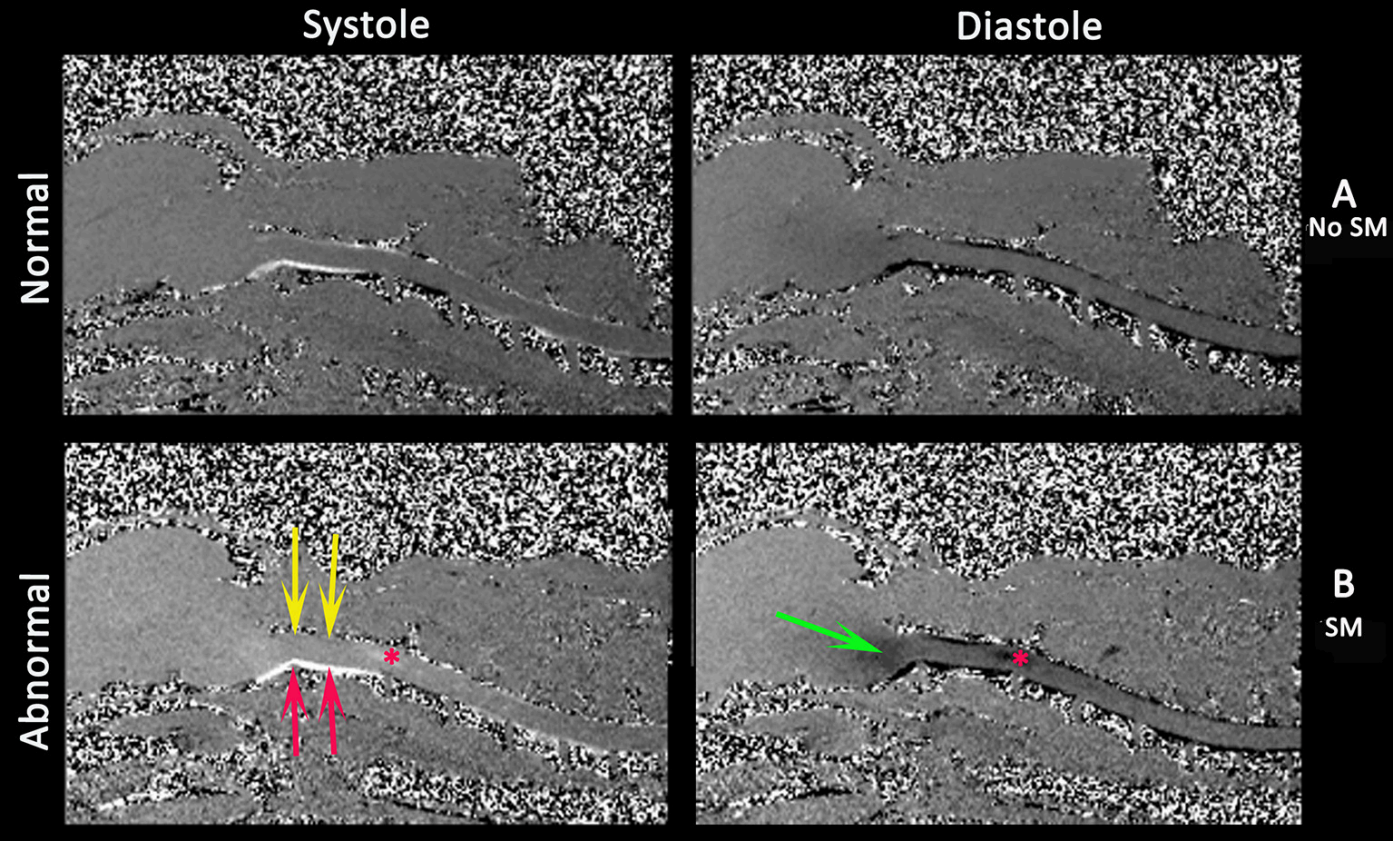
Phase contrast cine MRI of the brain and cervical spinal cord in normal **(A)** and a CMSM affected CKCS **(B)**. Left systole, right diastole. Compared to normal **(A)**, the SM affected **(B)** has little caudal flow in the dorsal cervical subarachnoid space (yellow arrows) and by contrast high velocity flow in the ventral cervical subarachnoid space (red arrows). There is pulsatile flow in the syrinx at the level of C2/C3 (asterisk) and fluid entering the medulla parenchyma (green arrows) (Siemens Symphony Tim system, 1.5 T, Enlangen Germany).

MRI flow studies are an integral part of the diagnostic work up of CM and SM in human patients (14, 102) and for review of the clinical application for SM, arachnoid webs and the subarachnoid space see Li et al. (104). Standard MRI sequences of the brain and spinal cord assume that the tissue is static with movement only occurring in blood vessels and CSF and the aim is to achieve higher spatial and contrast resolution typically at the expense of temporal resolution. This results in motion-related blurring of non-static structures (104). Cine MRI uses cardiac gating using electrocardiogram or pulse oximetry. Phase-contrast cine MRI (Figure 7) measures pulsatile CSF motion influenced by the cardiac cycle and can measure both CSF and syrinx fluid velocities in a defined area of interest (102, 127). By contrast cine bSSFP allows appreciation of the movement of the central nervous system and structures within the subarachnoid space. The data for a single slice is acquired multiple times over the cardiac cycle with each single image corresponding to single point in that cycle (termed a cardiac phase). All the images are then viewed sequentially as a cine loop (104). Phase contrast cine MRI is used to localize CSF channel obstruction, to demonstrate improvement (or lack thereof) post operatively and also has a role in prognostication. Syrinx fluid movement, as detected by phase contrast cine MRI, is associated with progressive neurological signs whereas lack of syrinx fluid movement is associated with no or stable neurological signs (102, 128). However, it does not allow visualization of arachnoid membranes and so in humans, and for investigation of idiopathic SM, Phase contrast cine MRI is used to localize the area of flow abnormalities/obstruction and cine bSSFP is used to define the arachnoid webs or bands (104).

In veterinary medicine, phase contrast cine MRI has been used to investigate CSF flow (80) and cine bSSFP used to investigate cerebellar movement (76) (Table 3) but its application as a diagnostic tool has yet to be realized.

To make a diagnosis of symptomatic CM and or SM the diagnostic imaging should be related to the clinical history and examination findings.

## Diagnosis of CM-pain

**Figure 8:**
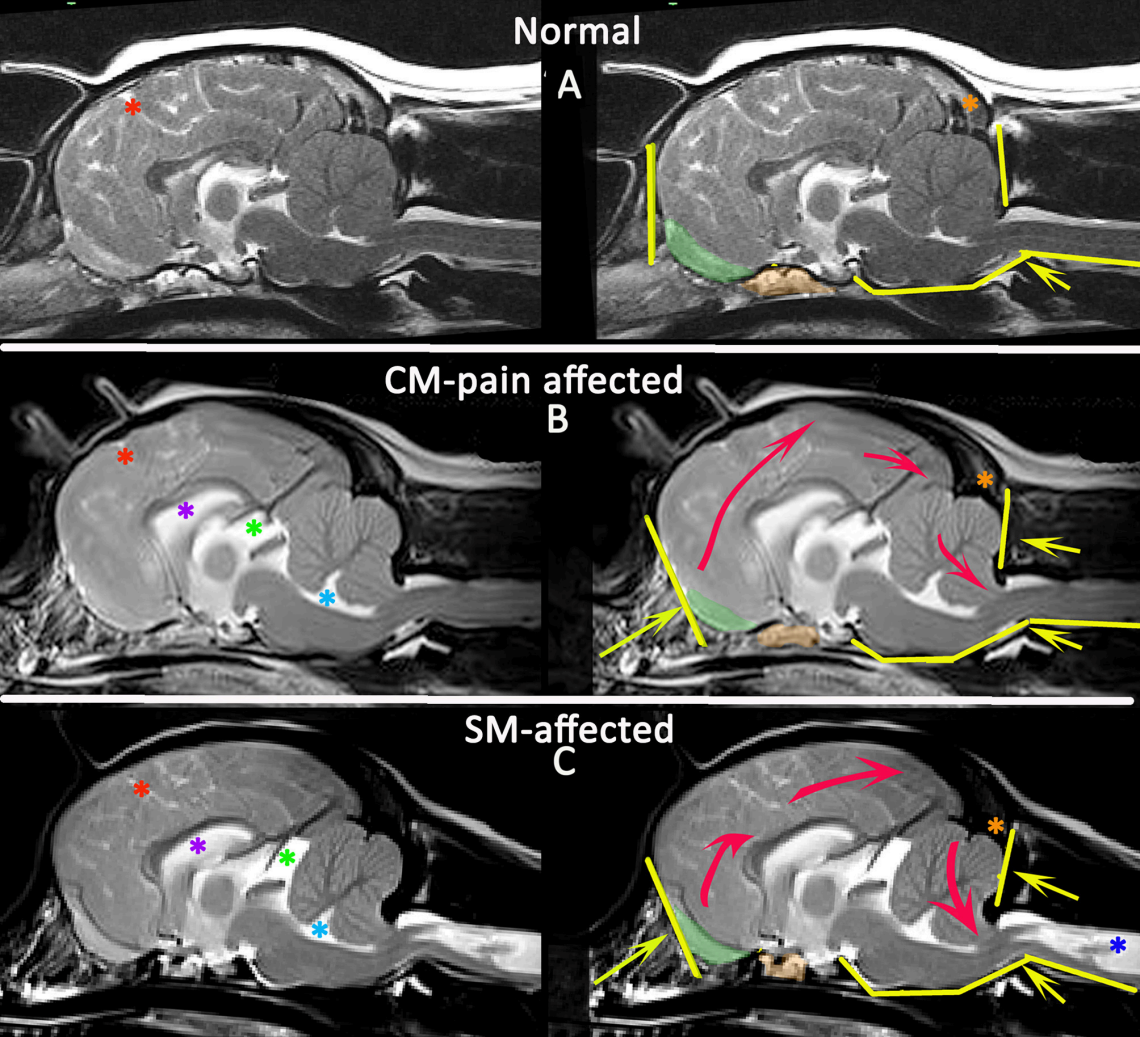
Morphometric changes in CMSM. Mid-sagittal T2-weighted brain MRI of three CKCS: normal **(A)**, CM-pain affected **(B)**, and SM-affected **(C)**. The left column compares the CSF spaces. There is effacement of the cranial subarachnoid spaces evidenced by reduced definition of the sulci filled with high signal CSF in affected dogs **(B,C)**. A Red asterisk highlights the cruciate sulcus for comparison. In addition there is ventriculomegaly with dilatation of the lateral ventricle (purple asterisk), third ventricle and quadrigeminal cistern (green asterisk) and fourth ventricle (aqua asterisk). The right column compares the neuro-anatomical changes and also the direction in which the neuroparenchyma has been deformed and crowded by the bony restrictions. Compared to the normal CKCS **(A)**, the affected CKCS **(B,C)** are more brachycephalic with shortening of the basicranium and prephenoid bone (orange shading) with reduced and more ventrally orientated olfactory bulbs (green shading). The rostral forebrain is flattened in **(B,C)** and the rostrotentorial neuroparenchyma is displaced dorsocaudally giving increased height to the cranium particularly in **(B)**. This reduces the functional caudotentorial space contributing to the hindbrain herniation. The atlas is closer to the skull and supraocciptal bone is flattened, particularly in **(C)**, the SM-affected dog which also has a reduction of the occipital crest (orange asterisk). In addition to the brachycephaly, the SM affected dog has further compromise of the craniocervical junction by a cervical flexure and acute angulation of the odontoid peg resulting in kinking/ elevation of the craniospinal junction. The syrinx is indicated by the dark blue asterisk (Siemens Magnetom Symphony, A Tim System, 1.5 T, Erlangen, Germany).

CM-pain is a difficult diagnosis because the clinical signs are non-specific and/or have alternative explanations but should be considered in a predisposed breed presenting with a signs suggesting pain such as; a history of vocalization described as without obvious trigger, when shifting position when recumbent and when being lifted under the sternum to a height; spinal pain; head and ear rubbing or scratching; refusal or difficulty jumping or doing stairs; exercise intolerance/reduced activity; sleep disruption; or behavioral change described as becoming more anxious, aggressive, or withdrawn (48). Morphometric studies found CM pain was associated with increasing brachycephaly with shortening of the skull base with rostrotentorial overcrowding resulting in rostral flattening of the forebrain, reduction and ventral displacement of the olfactory bulbs and increased height of the cranium, especially in the occipital region (39). There may be changes suggesting obstruction of CSF channels including reduction in the cranial and spinal subarachnoid space in addition to ventriculomegaly of all ventricles and cisterns except the cisterna magna which is often reduced (Figure 7). The craniocervical changes are less pronounced than in SM affected dogs and indeed this feature is hypothesized to protect this cohort from developing SM (39). Ultimately the diagnosis must be made by ruling out other causes of pain in combination with consistent clinical and MRI findings. A guide is suggested in Figures 5, 8.

## Diagnosis of symptomatic SM

**Figure 9:**
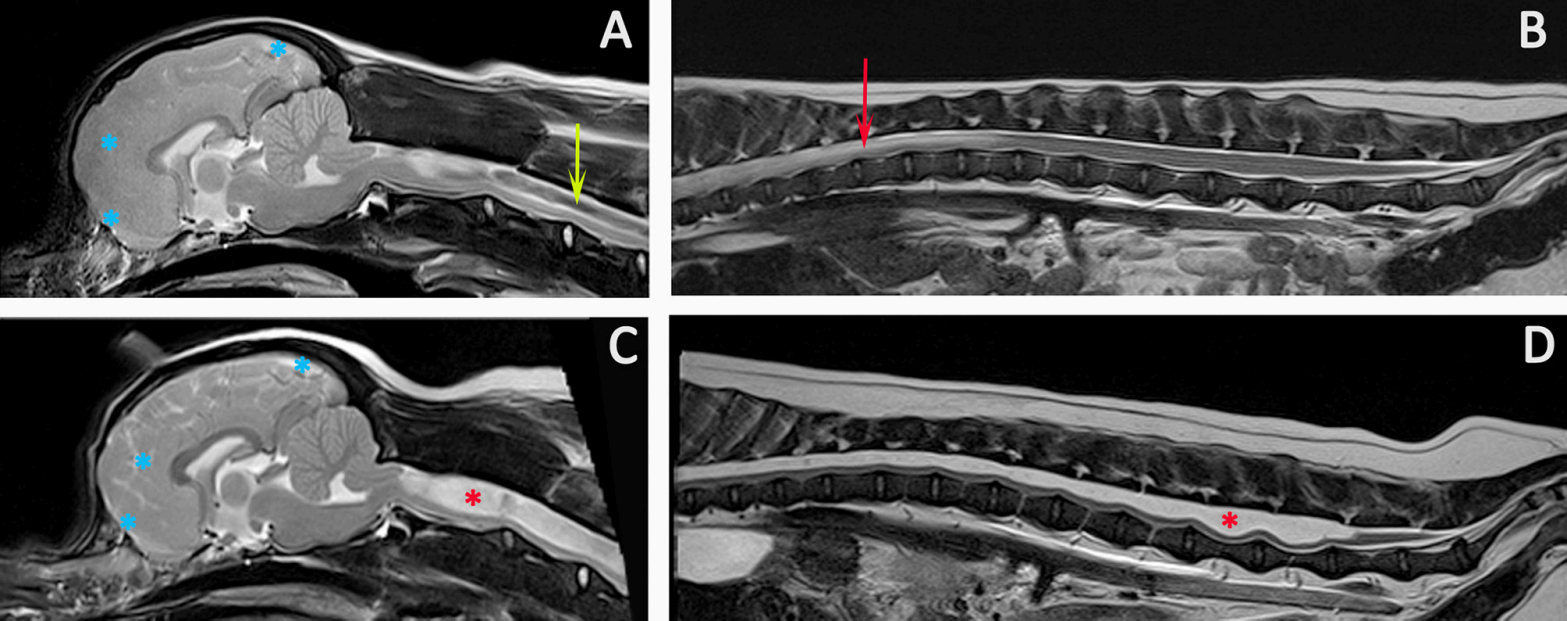
Progression of SM. Diagnostic Imaging of a female CKCS at 8 months old **(A,B)** and 3 years old **(C,D)**. The CKCS was originally presented with signs of pain. When represented at 3 years of age, the signs of pain were no longer controlled by medication and she had mild thoracic limb hypermetria, tetraparesis, and pelvic limb ataxia. T2-weighted mid-sagittal MRI of the brain and cranial cervical spinal cord in a CM and SM affected CKCS **(A)** 8 months, **(C)** 3 years old T2-weighted mid-sagittal MRI of the vertebral column from T7 to Cy1 **(B)** 8 months, **(D)** 3 years old. **(A,B)** Imaging indicated risk of progressive SM with loss of the cranial subarachnoid space and effacement of the cranial sulci (blue asterisks), fluid signal-void sign within the syrinx suggesting pulsatile or turbulent flow (green arrow) and a pre-syrinx (oedema) and central canal dilatation in the caudal thoracic spinal cord (red arrow). **(C,D)** The syrinx has progressed to holocord SM (red asterisk). Although conclusions cannot be drawn without pressure measurements, it is interesting that MRI signs that can suggest elevated intra-cranial pressure (129) and fluid surging and pulsation within the syrinx (117) are diminished with an appreciably greater cranial subarachnoid space indicated by high signal CSF within the sulci (blue asterisks) and less fluid signal-void sign within the syrinx (Siemens Magnetom Symphony, A Tim System, 1.5 T, Erlangen, Germany).

The diagnosis of SM implies a fluid filled cavity related to disturbance of CSF flow, spinal cord tethering or intramedullary tumor; it is not an appropriate description for myelomalacia or cystic lesions (14). SM can be asymptomatic (34) and when interpreting MRI, an assessment should be made as to whether the location and severity of the syrinx would account for the signs. Signs of an “active” and filling syrinx include fluid signal-void sign within the syrinx cavity indicating pulsatile or turbulent flow, hypothesized to be a cause of syrinx propagation (117) (Figure 9).

In addition an active and filling syrinx is expansive within the spinal cord and generally has an asymmetrical shape on transverse images. By contrast a quiescent syrinx is centrally located, elliptical on sagittal images and symmetrical, usually circular on transverse images and results in little or no change to the outline of the spinal cord (87, 130). Symptomatic SM results in a myelopathy and therefore is suggested by sensory and motor signs that localize to the level of the spinal cord affected by the syrinx. However, gait disturbances may be surprisingly mild even with extensive SM. The dog in Figure 9 was presented with a mild pelvic limb ataxia, thoracic limb hypermetria and tetraparesis and was considered by the owner to have a normal exercise tolerance. If the clinical signs are disproportionate to the extent of the syrinx, other differentials should be explored for example degenerative myelopathy related to superoxide dismutase-1 mutation as a cause of pelvic limb paresis (48, 131). The maximum width of the syrinx on transverse images should be assessed; myelopathic signs in CKCS are associated with a syrinx transverse width of 4 mm or more whereas phantom scratching and cervicotorticollis/scoliosis are associated with extension of the syrinx into the superficial dorsal horn ipsilateral to the phantom scratching side and/or contralateral to the head tilt (48, 87). Phantom scratching is associated with extension to the superficial dorsal horn ipsilateral to the scratching side in the C3-C6 spinal cord segments (corresponding to C2-C5 vertebrae) (88). A guide is suggested in Figures 5, 8.

## Conclusion

Diagnosis of CM-pain is made by appropriate clinical signs in addition to MRI brain findings of a brachycephaly with rostrotentorial crowding including rostral flattening, olfactory bulb reduction and rotation, increased height of the cranium with reduction of the functional caudotentorial space and hindbrain herniation. There may also be changes suggesting raised intracranial pressure such as loss of sulci definition with ventriculomegaly. The cisterna magna is reduced. In addition to these brachycephalic changes, dogs with SM are more likely to have craniocervical junction abnormalities including rostral displacement of the axis and atlas with increased odontoid angulation causing craniospinal deformation and medullary oblongata elevation. Symptomatic SM is diagnosed on the basis of signs of myelopathy and presence of a large syrinx that is consistent with the neuro-localization. The imaging protocol should establish the longitudinal and transverse extent of the syrinx. If the cause of CSF channel disruption is not revealed by anatomical MRI then other imaging techniques such as bSSFP sequences may be appropriate.

## Author contributions

CR: concept and original draft. FS: editing and reviewing draft. SK: graphics, editing, and reviewing draft.

### Conflict of interest statement

The authors declare that the research was conducted in the absence of any commercial or financial relationships that could be construed as a potential conflict of interest.

## Supplementary material

Supplementary Table 1 MRI parameters for a CM and SM specific protocol on a 1.5T machine.

Supplementary Figure 1 Duplicate of Figure 5 with a higher resolution.

## References

[1] Knowler SP Galea GL Rusbridge C. Morphogenesis of canine chiari malformation and secondary syringomyelia: disorders of cerebrospinal fluid circulation. Front Vet Sci. (2018) 5:171. 10.3389/fvets.2018.00171 30101146 PMC6074093

[2] Cappello R Rusbridge C. Report from the Chiari-Like Malformation and Syringomyelia Working Group round table. Vet Surg. (2007) 36:509–12. 10.1111/j.1532-950X.2007.00298.x 17614933

[3] Chiari H Ueber Veränderungen des Kleinhirns infolge von Hydrocephalie des Grosshirns. Dtsch Med Wochenschir. (1891) 42:1172–5. 10.1055/s-0029-1206803

[4] Rusbridge C Flint G (eds). Nomenclature. In: Syringomyelia: A Disorder of CSF Circulation. Berlin; Heidelberg: Springer (2014).p. 301–9. 10.1007/978-3-642-13706-8_20

[5] Chiari H Ueber Veränderungen des Kleinhirns, des Pons and der medulla oblongata in Folge von genitaler Hydrocephalie des Grosshirns. Denkschr Akad Wiss Wien (1896) 63:71–116.

[6] Azahraa Haddad F Qaisi I Joudeh N Dajani H Jumah F Elmashala A. The newer classifications of the chiari malformations with clarifications: an anatomical review. Clin Anat. (2018) 31:314–22. 10.1002/ca.23051 29344999

[7] Milhorat TH Chou MW Trinidad EM Kula RW Mandell M Wolpert C. Chiari I malformation redefined: clinical and radiographic findings for 364 symptomatic patients. Neurosurgery (1999) 44:1005–17. 10.1097/00006123-199905000-00042 10232534

[8] Cinalli G Spennato P Sainte-Rose C Arnaud E Aliberti F Brunelle F. Chiari malformation in craniosynostosis. Childs Nerv Syst. (2005) 21:889–901. 10.1007/s00381-004-1115-z 15875201

[9] Milhorat TH Bolognese PA Nishikawa M Mcdonnell NB Francomano CA. Syndrome of occipitoatlantoaxial hypermobility, cranial settling, and chiari malformation type I in patients with hereditary disorders of connective tissue. J Neurosurg Spine (2007) 7:601–9. 10.3171/SPI-07/12/601 18074684

[10] Milhorat TH Nishikawa M Kula RW Dlugacz YD. Mechanisms of cerebellar tonsil herniation in patients with Chiari malformations as guide to clinical management. Acta Neurochir. (2010) 152:1117–27. 10.1007/s00701-010-0636-3 20440631 PMC2887504

[11] Bejjani GK. Association of the Adult Chiari Malformation and Idiopathic Intracranial Hypertension: more than a coincidence. Med Hypotheses (2003) 60:859–63. 10.1016/S0306-9877(03)00064-1 12699714

[12] Richard S Humbertjean L Mione G Braun M Schmitt E Colnat-Coulbois S. Syringomyelia caused by traumatic intracranial hypotension: case report and literature review. World Neurosurg. (2016) 91:674–8 e613. 10.1016/j.wneu.2016.04.062 27126910

[13] Brodbelt A The Biochemistry of Syringomyelia. In: Flint G Rusbridge C, editors. Syringomyelia: A Disorder of CSF Circulation. Berlin; Heidelberg: Springer (2014).p. 261–78.

[14] Klekamp J. How should syringomyelia be defined and diagnosed? World Neurosurg. (2018) 111:e729–e745. 10.1016/j.wneu.2017.12.156 29317358

[15] Da Costa RC Parent JM Poma R Duque MC. Cervical syringohydromyelia secondary to a brainstem tumor in a dog. J Am Vet Med Assoc. (2004) 225:1061–64, 1048. 10.2460/javma.2004.225.1061 15515984

[16] Jung DI Park C Kang BT Kim JW Kim HJ Lim CY. Acquired cervical syringomyelia secondary to a brainstem meningioma in a maltese dog. J Vet Med Sci. (2006) 68:1235–8. 10.1292/jvms.68.1235 17146188

[17] Mackillop E Schatzberg SJ De Lahunta A. Intracranial epidermoid cyst and syringohydromyelia in a dog. Vet Radiol Ultrasound (2006) 47:339–44. 10.1111/j.1740-8261.2006.00150.x 16863050

[18] Szabo D Saveraid TC Rodenas S. Cervicothoracic syringohydromyelia associated with a prosencephalic mass in a dog. J Small Anim Pract. (2012) 53:613–7. 10.1111/j.1748-5827.2012.01266.x 23013378

[19] Oxley W Pink J. Amelioration of caudal thoracic syringohydromyelia following surgical management of an adjacent arachnoid cyst. J Small Anim Pract. (2012) 53:67–72. 10.1111/j.1748-5827.2011.01146.x 22122126

[20] Rohdin C Nyman HT Wohlsein P Hultin Jaderlund K. Cervical spinal intradural arachnoid cysts in related, young pugs. J Small Anim Pract. (2014) 55:229–34. 10.1111/jsap.12167 24372140

[21] Kiviranta AM Lappalainen AK Hagner K Jokinen T. Dermoid sinus and spina bifida in three dogs and a cat. J Small Anim Pract. (2011) 52:319–24. 10.1111/j.1748-5827.2011.01062.x 21627660

[22] Ollivier D'angers C-P De la Moelle Épinière et de Ses Maladies. Paris: Crevot (1824).

[23] Ollivier D'angers C-P Traité des Maladies de la Moelle Épinière Contenant l'Histoire Anatomique, Physiologique et Pathologique de ce Centre Nerveux Chez l'Homme. Paris: Crevot (1827).

[24] Schuppel Über Hydromyelus. Arch Heilk (1865) 6:289.

[25] Simon T Über Syringomyelia und Geshwulstbildung im Rückenmark. Arch. Psychiat. NervenKrank (1875) 5, 120–163.

[26] Newton EJ. Syringomyelia as a manifestation of defective fourth ventricular drainage. Ann R Coll Surg Engl. (1969) 44:194–213. 5305109 PMC2387612

[27] Leyden E Über Hydromyelus un Syringomyelie. Arch Pathol Anat Physiol. (1876) 68:1–20. 10.1007/BF01879533

[28] Kahler O Pick A Beitrag zur Lehre von der Syringo-und Hydromyelia. Vjschr Prakt Heilkd. (1879) 142:20–41.

[29] Hogg JP Peterson AM El-Kadi M Imaging of cranial and spinal cerebrospinal fluid collections. In: Kaufman HH, editor. Cerebrospinal Fluid Collections. San Francisco, CA: American Association of Neurological Surgeons (1998), pp. 19–56.

[30] Chiari H Ueber die Pathogenese der Sogenannten Syringomyelie. (Hierzu Tafal 13.). Zeitschrift für Heilkunde (1888) 9, 307.

[31] Radojicic M Nistor G Keirstead HS. Ascending central canal dilation and progressive ependymal disruption in a contusion model of rodent chronic spinal cord injury. BMC Neurol (2007) 7:30. 10.1186/1471-2377-7-30 17822568 PMC2018707

[32] Hu HZ Rusbridge C Constantino-Casas F Jeffery N. Histopathological investigation of syringomyelia in the Cavalier King Charles Spaniel. J Comp Pathol. (2012) 146:192–201. 10.1016/j.jcpa.2011.07.002 21889166

[33] Bva T Chiari Malformation/Syringomyelia Scheme (CM/SM Scheme) [Online] (2013). Available online at: http://www.bva.co.uk/canine_health_schemes/ChiariMalformationSyringomyeliaSchemeCMSMScheme.aspx: British Veterinary Assocication (Accessed July, 8 2013).

[34] Parker JE Knowler SP Rusbridge C Noorman E Jeffery ND. Prevalence of asymptomatic syringomyelia in Cavalier King Charles spaniels. Vet Rec. (2011) 168:667. 10.1136/vr.d1726 21672954

[35] Wijnrocx K Van Bruggen LWL Eggelmeijer W Noorman E Jacques A Buys N. Twelve years of chiari-like malformation and syringomyelia scanning in Cavalier King Charles Spaniels in the Netherlands: towards a more precise phenotype. PLoS ONE (2017) 12:e0184893. 10.1371/journal.pone.0184893 28934242 PMC5608246

[36] Rusbridge C Chiari–like malformation and syringomyelia. Eur J Compan Anim. Pract (2013) 23:70–89. Available online at: http://www.ejcap.org/issues/ejcap-233-autumn-2013/

[37] Sparks CR Cerda-Gonzalez S Griffith EH Lascelles BDX Olby NJ. Questionnaire-based analysis of owner-reported scratching and pain signs in cavalier king charles spaniels screened for chiari-like malformation and syringomyelia. J Vet Intern Med. (2018) 32:331–9. 10.1111/jvim.14856 29105875 PMC5787193

[38] Knowler SP Mcfadyen AK Freeman C Kent M Platt SR Kibar Z. Quantitative analysis of Chiari-like malformation and syringomyelia in the griffon bruxellois dog. PLoS ONE (2014) 9:e88120. 10.1371/journal.pone.0088120 24533070 PMC3922758

[39] Knowler SP Cross C Griffiths S Mcfadyen AK Jovanovik J Tauro A. Use of morphometric mapping to characterise symptomatic chiari-like malformation, secondary syringomyelia and associated brachycephaly in the cavalier king charles spaniel. PLoS ONE (2017) 12:e0170315. 10.1371/journal.pone.0170315 28122014 PMC5266281

[40] Knowler SP Kiviranta AM Mcfadyen AK Jokinen TS La Ragione RM Rusbridge C. Craniometric analysis of the hindbrain and craniocervical junction of chihuahua, affenpinscher and cavalier king charles spaniel dogs with and without syringomyelia secondary to chiari-like malformation. PLoS ONE (2017) 12:e0169898. 10.1371/journal.pone.0169898 28121988 PMC5266279

[41] Lemay P Knowler SP Bouasker S Nedelec Y Platt S Freeman C. Quantitative Trait Loci (QTL) study identifies novel genomic regions associated to chiari-like malformation in griffon bruxellois dogs. PLoS ONE (2014) 9:e89816. 10.1371/journal.pone.0089816 24740420 PMC3989173

[42] Ancot F Lemay P Knowler SP Kennedy K Griffiths S Cherubini GB. A genome-wide association study identifies candidate loci associated to syringomyelia secondary to Chiari-like malformation in Cavalier King Charles Spaniels. BMC Genet (2018) 19:16. 10.1186/s12863-018-0605-z 29566674 PMC5865342

[43] Spiteri M Knowler SP Wells K Rusbridge C Mapping morphological change in cavalier king charles spaniels with syringomyelia using novel machine learning approach. In: 30th Symposium ESVN-ECVN, Helsinki (2017).

[44] Spiteri M Knowler SP Wells K Rusbridge C Mapping Morphological Change in Cavalier King Charles Spaniels With Chiari-like malformation associated pain. In: 30th Symposium ESVN-ECVN, Helsinki (2017).

[45] Thofner MS Stougaard CL Westrup U Madry AA Knudsen CS Berg H. Prevalence and heritability of symptomatic syringomyelia in Cavalier King Charles Spaniels and long-term outcome in symptomatic and asymptomatic littermates. J Vet Intern Med. (2015) 29:243–50. 10.1111/jvim.12475 25308931 PMC4858089

[46] Schulze S Refai M Deutschland M Failing K Schmidt M. Prevalence of syringomyelia in clinically unaffected Cavalier King Charles Spaniels in Germany (2006-2016). Tierarztl Prax Ausg K Kleintiere Heimtiere (2018) 46:157–62. 10.15654/TPK-170725 29898477

[47] Kiviranta AM Rusbridge C Laitinen-Vapaavuori O Hielm-Bjorkman A Lappalainen AK Knowler SP. Syringomyelia and Craniocervical Junction Abnormalities in Chihuahuas. J Vet Intern Med. (2017) 31:1771–81. 10.1111/jvim.14826 28892202 PMC5697179

[48] Rusbridge C Behavioural and clinical signs of Chiari-like malformation and syringomyelia in Cavalier King Charles spaniels. In: Murphy S, editor. BSAVA Congress 2018. Birmingham: British Small Animal Veterinary Association (2018). p. 430.

[49] Stockyard CR The genetic and endocrinic basis for differences in form and behaviour. In: Anatomical Memoirs (Philadelphia, PA: Wistar Institute of Anatomy and Biology) (1941).p. 340–57.

[50] Schmidt MJ Volk H Klingler M Failing K Kramer M Ondreka N. Comparison of closure times for cranial base synchondroses in mesaticephalic, brachycephalic, and cavalier king charles spaniel dogs. Vet Radiol Ultrasound (2013) 54:497–503. 10.1111/vru.12072 23782353

[51] Schmidt MJ Neumann AC Amort KH Failing K Kramer M. Cephalometric measurements and determination of general skull type of Cavalier King Charles Spaniels. Vet Radiol Ultrasound (2011) 52:436–40. 10.1111/j.1740-8261.2011.01825.x 21521397

[52] Rusbridge C Knowler SP Pieterse L Mcfadyen AK. Chiari-like malformation in the Griffon Bruxellois. J Small Anim Pract. (2009) 50:386–93. 10.1111/j.1748-5827.2009.00744.x 19689665

[53] Mitchell TJ Knowler SP Van Den Berg H Sykes J Rusbridge C. Syringomyelia: determining risk and protective factors in the conformation of the Cavalier King Charles Spaniel dog. Canine Genet Epidemiol. (2014) 1:9. 10.1186/2052-6687-1-9 26401326 PMC4579371

[54] Dubrul EL Laskin DM. Preadaptive potentialities of the mammalian skull: an experiment in growth and form. Am J Anat. (1961) 109:117–32. 10.1002/aja.1001090203 13888118

[55] Scrivani PV Thompson MS Winegardner KR Dewey CW Scarlett JM. Association between frontal-sinus size and syringohydromyelia in small-breed dogs. Am J Vet Res. (2007) 68:610–3. 10.2460/ajvr.68.6.610 17542693

[56] Carrera I Dennis R Mellor DJ Penderis J Sullivan M. Use of magnetic resonance imaging for morphometric analysis of the caudal cranial fossa in Cavalier King Charles Spaniels. Am J Vet Res. (2009) 70:340–5. 10.2460/ajvr.70.3.340 19254145

[57] Driver CJ Rusbridge C Cross HR Mcgonnell I Volk HA. Relationship of brain parenchyma within the caudal cranial fossa and ventricle size to syringomyelia in cavalier King Charles spaniels. J Small Anim Pract. (2010) 51:382–6. 10.1111/j.1748-5827.2010.00952.x 20536691

[58] Shaw TA Mcgonnell IM Driver CJ Rusbridge C Volk HA. Caudal cranial fossa partitioning in Cavalier King Charles spaniels. Vet Rec. (2013) 172:341. 10.1136/vr.101082 23385008

[59] Schmidt MJ Kramer M Ondreka N. Comparison of the relative occipital bone volume between Cavalier King Charles spaniels with and without syringohydromyelia and French bulldogs. Vet Radiol Ultrasound (2012) 53:540–4. 10.1111/j.1740-8261.2012.01955.x 22702890

[60] Schmidt MJ Ondreka N Rummel C Volk H Sauerbrey M Kramer M. Volume reduction of the jugular foramina in Cavalier King Charles Spaniels with syringomyelia. BMC Vet Res. (2012) 8:158. 10.1186/1746-6148-8-158 22954070 PMC3514347

[61] Fenn J Schmidt MJ Simpson H Driver CJ Volk HA. Venous sinus volume in the caudal cranial fossa in Cavalier King Charles spaniels with syringomyelia. Vet J. (2013) 197:896–7. 10.1016/j.tvjl.2013.05.007 23755937

[62] Cerda-Gonzalez S Olby NJ Mccullough S Pease AP Broadstone R Osborne JA. Morphology of the caudal fossa in Cavalier King Charles Spaniels. Vet Radiol Ultrasound (2009) 50:37–46. 10.1111/j.1740-8261.2008.01487.x 19241752

[63] Marino DJ Loughin CA Dewey CW Marino LJ Sackman JJ Lesser ML. Morphometric features of the craniocervical junction region in dogs with suspected Chiari-like malformation determined by combined use of magnetic resonance imaging and computed tomography. Am J Vet Res. (2012) 73:105–11. 10.2460/ajvr.73.1.105 22204295

[64] Cerda-Gonzalez S Bibi KF Gifford AT Mudrak EL Scrivani PV. Magnetic resonance imaging-based measures of atlas position: Relationship to canine atlantooccipital overlapping, syringomyelia and clinical signs. Vet J. (2016) 209:133–8. 10.1016/j.tvjl.2016.01.008 26857868

[65] Cerda-Gonzalez S Dewey CW Scrivani PV Kline KL. Imaging features of atlanto-occipital overlapping in dogs. Vet Radiol Ultrasound (2009) 50:264–8. 10.1111/j.1740-8261.2009.01531.x 19507388

[66] Cerda-Gonzalez S Olby NJ Griffith EH. Dorsal compressive atlantoaxial bands and the craniocervical junction syndrome: association with clinical signs and syringomyelia in mature cavalier king charles spaniels. J Vet Intern Med. (2015) 29:887–92. 10.1111/jvim.12604 25996662 PMC4895407

[67] Carruthers H Rusbridge C Dube MP Holmes M Jeffery N. Association between cervical and intracranial dimensions and syringomyelia in the cavalier King Charles spaniel. J Small Anim Pract. (2009) 50:394–8. 10.1111/j.1748-5827.2009.00768.x 19689666

[68] Stalin CE Rusbridge C Granger N Jeffery ND. Radiographic morphology of the cranial portion of the cervical vertebral column in Cavalier King Charles Spaniels and its relationship to syringomyelia. Am J Vet Res. (2008) 69:89–93. 10.2460/ajvr.69.1.89 18167092

[69] Cross HR Cappello R Rusbridge C. Comparison of cerebral cranium volumes between cavalier King Charles spaniels with Chiari-like malformation, small breed dogs and Labradors. J Small Anim Pract. (2009) 50:399–405. 10.1111/j.1748-5827.2009.00799.x 19689667

[70] Driver CJ Rusbridge C Cross HR Mcgonnell I Volk HA Association between Chiari-like malformation and syringomyelia in cavalier King Charles spaniels. Vet Rec. (2010) 167:306 10.1136/vr.167.8.306-a 21262715

[71] Shaw TA Mcgonnell IM Driver CJ Rusbridge C Volk HA. Increase in cerebellar volume in Cavalier King Charles Spaniels with Chiari-like malformation and its role in the development of syringomyelia. PLoS ONE (2012) 7:e33660. 10.1371/journal.pone.0033660 22506005 PMC3323625

[72] Lu D Lamb CR Pfeiffer DU Targett MP. Neurological signs and results of magnetic resonance imaging in 40 cavalier King Charles spaniels with Chiari type 1-like malformations. Vet Rec. (2003) 153:260–3. 10.1136/vr.153.9.260 12974337

[73] Harcourt-Brown TR Campbell J Warren-Smith C Jeffery ND Granger NP. Prevalence of Chiari-like malformations in clinically unaffected dogs. J Vet Intern Med. (2015) 29:231–7. 10.1111/jvim.12477 25319206 PMC4858087

[74] Rusbridge C Knowler SP. Coexistence of occipital dysplasia and occipital hypoplasia/syringomyelia in the cavalier King Charles spaniel. J Small Anim Pract. (2006) 47:603–6. 10.1111/j.1748-5827.2006.00048.x 17004953

[75] Driver CJ De Risio L Hamilton S Rusbridge C Dennis R Mcgonnell IM. Changes over time in craniocerebral morphology and syringomyelia in cavalier King Charles spaniels with Chiari-like malformation. BMC Vet Res. (2012) 8:215. 10.1186/1746-6148-8-215 23136935 PMC3514376

[76] Driver CJ Watts V Bunck AC Van Ham LM Volk HA. Assessment of cerebellar pulsation in dogs with and without Chiari-like malformation and syringomyelia using cardiac-gated cine magnetic resonance imaging. Vet J. (2013) 198:88–91. 10.1016/j.tvjl.2013.05.017 23770398

[77] Clarke EC Fletcher DF Stoodley MA Bilston LE. Computational fluid dynamics modelling of cerebrospinal fluid pressure in Chiari malformation and syringomyelia. J Biomech. (2013) 46:1801–9. 10.1016/j.jbiomech.2013.05.013 23769174

[78] Clarke EC Stoodley MA Bilston LE. Changes in temporal flow characteristics of CSF in Chiari malformation Type I with and without syringomyelia: implications for theory of syrinx development. J Neurosurg. (2013) 118:1135–40. 10.3171/2013.2.JNS12759 23495878

[79] Cerda-Gonzalez S Olby NJ Griffith EH. Medullary position at the craniocervical junction in mature cavalier king charles spaniels: relationship with neurologic signs and syringomyelia. J Vet Intern Med. (2015) 29:882–6. 10.1111/jvim.12605 25929341 PMC4895408

[80] Cerda-Gonzalez S Olby NJ Broadstone R Mccullough S Osborne JA. Characteristics of cerebrospinal fluid flow in Cavalier King Charles Spaniels analyzed using phase velocity cine magnetic resonance imaging. Vet Radiol Ultrasound (2009) 50:467–76. 10.1111/j.1740-8261.2009.01571.x 19788029

[81] Scrivani PV Freer SR Dewey CW Cerda-Gonzalez S Cerebrospinal fluid signal-void sign in dogs. Vet Radiol Ultrasound (2009) 50:269–75. 10.1111/j.1740-8261.2009.01532.x 19507389

[82] Driver CJ Chandler K Walmsley G Shihab N Volk HA. The association between Chiari-like malformation, ventriculomegaly and seizures in cavalier King Charles spaniels. Vet J. (2012) 195:235–7. 10.1016/j.tvjl.2012.05.014 22749114

[83] Ives EJ Doyle L Holmes M Williams TL Vanhaesebrouck AE. Association between the findings on magnetic resonance imaging screening for syringomyelia in asymptomatic Cavalier King Charles spaniels and observation of clinical signs consistent with syringomyelia in later life. Vet J. (2015) 203:129–30. 10.1016/j.tvjl.2014.11.010 25475164

[84] Rusbridge C Greitz D Iskandar BJ. Syringomyelia: current concepts in pathogenesis, diagnosis, and treatment. J Vet Intern Med. (2006) 20:469–79. 10.1111/j.1939-1676.2006.tb02884.x 16734077

[85] Loderstedt S Benigni L Chandler K Cardwell JM Rusbridge C Lamb CR. Distribution of syringomyelia along the entire spinal cord in clinically affected Cavalier King Charles Spaniels. Vet J. (2011) 190:359–63. 10.1016/j.tvjl.2010.12.002 21216639

[86] Cirovic S Lloyd R Jovanovik J Volk HA Rusbridge C. Computer simulation of syringomyelia in dogs. BMC Vet Res. (2018) 14:82. 10.1186/s12917-018-1410-7 29523203 PMC5845370

[87] Rusbridge C Carruthers H Dube MP Holmes M Jeffery ND. Syringomyelia in cavalier King Charles spaniels: the relationship between syrinx dimensions and pain. J Small Anim Pract. (2007) 48:432–6. 10.1111/j.1748-5827.2007.00344.x 17608656

[88] Nalborczyk ZR Mcfadyen AK Jovanovik J Tauro A Driver CJ Fitzpatrick N. MRI characteristics for “phantom” scratching in canine syringomyelia. BMC Vet Res. (2017) 13:340. 10.1186/s12917-017-1258-2 29145838 PMC5691609

[89] Van Biervliet J De Lahunta A Ennulat D Oglesbee M Summers B. Acquired cervical scoliosis in six horses associated with dorsal grey column chronic myelitis. Equine Vet J. (2004) 36:86–92. 10.2746/0425164044864624 14756379

[90] Johanson CE Duncan JA III Klinge PM Brinker T Stopa EG Silverberg GD. Multiplicity of cerebrospinal fluid functions: new challenges in health and disease. Cerebrospinal Fluid Res. (2008) 5:10. 10.1186/1743-8454-5-10 18479516 PMC2412840

[91] Driver CJ Rusbridge C Mcgonnell IM Volk HA. Morphometric assessment of cranial volumes in age-matched Cavalier King Charles spaniels with and without syringomyelia. Vet Rec. (2010) 167:978–9. 10.1136/vr.c4109 21262715

[92] Tamburrini G Caldarelli M Massimi L Gasparini G Pelo S Di Rocco C. Complex craniosynostoses: a review of the prominent clinical features and the related management strategies. Childs Nerv Syst. (2012) 28:1511–23. 10.1007/s00381-012-1819-4 22872268

[93] Baig MN Byrne F Devitt A Mccabe JP. Signs of nature in spine radiology. Cureus (2018) 10:e2456. 10.7759/cureus.2456 29888160 PMC5991933

[94] Unsgard G Solheim O Lindseth F Selbekk T. Intra-operative imaging with 3D ultrasound in neurosurgery. Acta Neurochir Suppl. (2011) 109:181–6. 10.1007/978-3-211-99651-5_28 20960340

[95] Narenthiran G Parks C Pettorini B. Management of Chiari I malformation in children: effectiveness of intra-operative ultrasound for tailoring foramen magnum decompression. Childs Nerv Syst. (2015) 31:1371–6. 10.1007/s00381-015-2699-1 25874846

[96] Schmidt MJ Wigger A Jawinski S Golla T Kramer M. Ultrasonographic appearance of the craniocervical junction in normal brachycephalic dogs and dogs with caudal occipital (Chiari-like) malformation. Vet Radiol Ultrasound (2008) 49:472–6. 10.1111/j.1740-8261.2008.00411.x 18833958

[97] Schmidt MJ Biel M Klumpp S Schneider M Kramer M. Evaluation of the volumes of cranial cavities in Cavalier King Charles Spaniels with Chiari-like malformation and other brachycephalic dogs as measured via computed tomography. Am J Vet Res. (2009) 70:508–12. 10.2460/ajvr.70.4.508 19335107

[98] Kromhout K Van Bree H Broeckx BJ Bhatti S Van Ham L Polis I. Low-field MRI and multislice CT for the detection of cerebellar (foramen magnum) herniation in Cavalier King Charles Spaniels. J Vet Intern Med. (2015) 29:238–42. 10.1111/jvim.12498 25408117 PMC4858103

[99] Garcia-Real I Kass PH Sturges BK Wisner ER. Morphometric analysis of the cranial cavity and caudal cranial fossa in the dog: a computerized tomographic study. Vet Radiol Ultrasound (2004) 45:38–45. 10.1111/j.1740-8261.2004.04006.x 15005359

[100] Galloway AM Curtis NC Sommerlad SF Watt PR. Correlative imaging findings in seven dogs and one cat with spinal arachnoid cysts. Vet Radiol Ultrasound (1999) 40:445–52. 10.1111/j.1740-8261.1999.tb00373.x 10528836

[101] Mauler DA De Decker S De Risio L Volk HA Dennis R Gielen I. Signalment, clinical presentation, and diagnostic findings in 122 dogs with spinal arachnoid diverticula. J Vet Intern Med. (2014) 28:175–81. 10.1111/jvim.12241 24428321 PMC4895525

[102] Heiss J Diagnostic Investigations. In: Flint G Rusbridge C, editors. Syringomyelia: A Disorder of CSF Circulation. Berlin; Heidelberg: Springer (2014).p. 125–40.

[103] Sayal PP Zafar A Carroll TA. Syringomyelia secondary to “occult” dorsal arachnoid webs: Report of two cases with review of literature. J Craniovertebr Junction Spine (2016) 7:101–4. 10.4103/0974-8237.181862 27217656 PMC4872557

[104] Li AE Wilkinson MD Mcgrillen KM Stoodley MA Magnussen JS. Clinical applications of cine balanced steady-state free precession MRI for the evaluation of the subarachnoid spaces. Clin Neuroradiol. (2015) 25:349–60. 10.1007/s00062-015-0383-1 25854921

[105] Goh S Bottrell CL Aiken AH Dillon WP Wu YW. Presyrinx in children with Chiari malformations. Neurology (2008) 71:351–6. 10.1212/01.wnl.0000304087.91204.95 18565831

[106] Bva Appendix 1 Breeding Recommendations Until Relevant EBVs are Available [Online] (2012). Available online at: http://www.bva.co.uk/public/documents/CM-SM_breeding_recommendations.pdf: BVA, The (Accessed July, 8 2013).

[107] Hecht S Huerta MM Reed RB. Magnetic resonance imaging (MRI) spinal cord and canal measurements in normal dogs. Anat Histol Embryol. (2014) 43:36–41. 10.1111/ahe.12045 23488993 PMC3933761

[108] Seo E Choi J Choi M Yoon J. Computed tomographic evaluation of cervical vertebral canal and spinal cord morphometry in normal dogs. J Vet Sci. (2014) 15:187–93. 10.4142/jvs.2014.15.2.187 24136210 PMC4087219

[109] Ricciardi M. Principles and applications of the balanced steady-state free precession sequence in small animal low-field MRI. Vet Res Commun. (2018) 42:65–86. 10.1007/s11259-017-9708-7 29302913

[110] Konar M Lang J. Pros and cons of low-field magnetic resonance imaging in veterinary practice. Vet Radiol Ultrasound (2011) 52:S5–14. 10.1111/j.1740-8261.2010.01780.x 21392156

[111] Oh CH Kim CG Lee JH Yoon SH Park HC Park CO. Missed diagnosis of syrinx. Asian Spine J. (2012) 6:1–5. 10.4184/asj.2012.6.1.1 22439081 PMC3302909

[112] Jinkins JR Sener RN. Idiopathic localized hydromyelia: dilatation of the central canal of the spinal cord of probable congenital origin. J Comput Assist Tomogr. (1999) 23:351–3. 10.1097/00004728-199905000-00004 10348436

[113] Upchurch JJ Mcgonnell IM Driver CJ Butler L Volk HA. Influence of head positioning on the assessment of Chiari-like malformation in Cavalier King Charles spaniels. Vet Rec. (2011) 169:277. 10.1136/vr.d4395 21824898

[114] Piesnack S Oechtering G Ludewig E. [Options for the reduction of magnetic susceptibility artifacts caused by implanted microchips in 0.5 Tesla magnetic resonance imaging]. Tierarztl Prax Ausg K Kleintiere Heimtiere (2015) 43:83–92. 10.15654/TPK-150048 25727725

[115] Dengg S Kneissl S. [Comparison of susceptibility artifacts generated by microchips with different geometry at 1.5 Tesla magnet resonance imaging. A phantom pilot study referring to the ASTM standard test method F2119-07]. Tierarztl Prax Ausg K Kleintiere Heimtiere (2013) 41:289–96. 10.1055/s-0038-1623723 24127025

[116] Ebraheim NA Savolaine ER Zeiss J Jackson WT. Titanium hip implants for improved magnetic resonance and computed tomography examinations. Clin Orthop Relat Res. (1992) 194–8. 10.1097/00003086-199202000-00028 1735213

[117] Sherman JL Barkovich AJ Citrin CM. The MR appearance of syringomyelia: new observations. AJR Am J Roentgenol. (1987) 148:381–91. 10.2214/ajr.148.2.381 3492118

[118] Ogbole GI Soneye MA Okorie CN Sammet S. Intraventricular cerebrospinal fluid pulsation artifacts on low-field magnetic resonance imaging: Potential pitfall in diagnosis? Niger Med J. (2016) 57:59–63. 10.4103/0300-1652.180565 27185981 PMC4859116

[119] Herlihy AH Oatridge A Curati WL Puri BK Bydder GM Hajnal JV. FLAIR imaging using nonselective inversion pulses combined with slice excitation order cycling and k-space reordering to reduce flow artifacts. Magn Reson Med. (2001) 46:354–64. 10.1002/mrm.1198 11477640

[120] Raybaud C Greenberg G Imaging (Normal and Abnormal). In: Mallucci C Sgouros S, editors. Cerebrospinal Fluid Disorders. Boca Raton, FL: CRC Press (2010).p. 66–109.

[121] Cherubini GB Platt SR Anderson TJ Rusbridge C Lorenzo V Mantis P. Characteristics of magnetic resonance images of granulomatous meningoencephalomyelitis in 11 dogs. Vet Rec. (2006) 159:110–5. 10.1136/vr.159.4.110 16861389

[122] Timpone VM Patel SH. MRI of a syrinx: is contrast material always necessary? AJR Am J Roentgenol. (2015) 204:1082–5. 10.2214/AJR.14.13310 25905945

[123] Kobayashi K Ando K Kato F Kanemura T Imagama S Sato K. MRI characteristics of spinal ependymoma in WHO Grade II: a review of 59 cases. Spine (2018) 43:E525–30. 10.1097/BRS.0000000000002496 29189641

[124] Tauro A Jovanovik J Driver CJ Rusbridge C. Clinical application of 3D-CISS MRI sequences for diagnosis and surgical planning of spinal arachnoid diverticula and adhesions in dogs. Vet Comp Orthop Traumatol. (2018) 31:83–94. 10.3415/VCOT-16-12-0169 29534275

[125] Roser F Ebner FH Danz S Riether F Ritz R Dietz K. Three-dimensional constructive interference in steady-state magnetic resonance imaging in syringomyelia: advantages over conventional imaging. J Neurosurg Spine (2008) 8:429–35. 10.3171/SPI/2008/8/5/429 18447688

[126] Seiler GS Robertson ID Mai W Widmer WR Suran J Nemanic S. Usefulness of a half-fourier acquisition single-shot turbo spin-echo pulse sequence in identifying arachnoid diverticula in dogs. Vet Radiol Ultrasound (2012) 53:157–61. 10.1111/j.1740-8261.2011.01893.x 22734150

[127] Battal B Kocaoglu M Bulakbasi N Husmen G Tuba Sanal H Tayfun C. Cerebrospinal fluid flow imaging by using phase-contrast MR technique. Br J Radiol. (2011) 84:758–65. 10.1259/bjr/66206791 21586507 PMC3473435

[128] Tobimatsu Y Nihei R Kimura T Suyama T Tobimatsu H. [A quantitative analysis of cerebrospinal fluid flow in posttraumatic syringomyelia]. Nihon Seikeigeka Gakkai Zasshi (1991) 65:505–16. 1835493

[129] Bittermann S Lang J Henke D Howard J Gorgas D. Magnetic resonance imaging signs of presumed elevated intracranial pressure in dogs. Vet J. (2014) 201:101–8. 10.1016/j.tvjl.2014.04.020 24888678

[130] Marks S Flint G Medicolegal Aspects. In: Flint G Rusbridge C, editors. Syringomyelia: A Disorder of CSF Circulation. Berlin; Heidelberg: Springer (2014).p. 289–300. 10.1007/978-3-642-13706-8_19

[131] Zeng R Coates JR Johnson GC Hansen L Awano T Kolicheski A. Breed distribution of SOD1 alleles previously associated with canine degenerative myelopathy. J Vet Intern Med. (2014) 28:515–21. 10.1111/jvim.12317 24524809 PMC4238831

