# Supplementary material for: Clinical Application of Diagnostic Imaging of Chiari-Like Malformation and Syringomyelia
Source: Front Vet Sci. 2018 Nov 28;5:280. doi: 10.3389/fvets.2018.00280 (PMC6279941; doi:10.3389/fvets.2018.00280)
Supplement: Supplementary Table 1 — MRI parameters for a CM and SM specific protocol on a 1.5T machine. [file Table_1.pdf]

SYRINGOMYELIA MRI PARAMETERS

ROUTINE

T2 Sagittal Brain	
Field Of View (mm)	170 x 170
Slice Thickness (mm)	2.7
Slice Gap (%)	20
Repetition Time (ms)	2380
Echo Time (ms)	99
Flip Angle (°)	150
Image Matrix	266 x 320
Bandwidth (Hz/Px)	80
Scan Time (min:sec)	4:35

T1 Sagittal Brain	
Field Of View (mm)	170 x 170
Slice Thickness (mm)	2.7
Slice Gap (%)	20
Repetition Time (ms)	400
Echo Time (ms)	14
Flip Angle (°)	80
Image Matrix	240 x 320
Bandwidth (Hz/Px)	100
Scan Time (min:sec)	4:52

T2 Transverse Brain	
Field Of View (mm)	140 x 98
Slice Thickness (mm)	3.5
Slice Gap (%)	10
Repetition Time (ms)	3470
Echo Time (ms)	95
Flip Angle (°)	150
Image Matrix	186 x 320
Bandwidth (Hz/Px)	65
Scan Time (min:sec)	4:08

T1 Transverse Brain	
Field Of View (mm)	140 x 98
Slice Thickness (mm)	3.5
Slice Gap (%)	10
Repetition Time (ms)	466
Echo Time (ms)	14
Flip Angle (°)	90
Image Matrix	157 x 320
Bandwidth (Hz/Px)	100
Scan Time (min:sec)	3:13

T2 Sagittal Spine	
Field Of View (mm)	260 x 260
Slice Thickness (mm)	1.5
Slice Gap (%)	10
Repetition Time (ms)	3990
Echo Time (ms)	105
Flip Angle (°)	180
Image Matrix	358 x 448
Bandwidth (Hz/Px)	151
Scan Time (min:sec)	5:09

T1 Sagittal Spine	
Field Of View (mm)	260 x 260
Slice Thickness (mm)	1.5
Slice Gap (%)	10
Repetition Time (ms)	450
Echo Time (ms)	12
Flip Angle (°)	150
Image Matrix	326 x 384
Bandwidth (Hz/Px)	191
Scan Time (min/sec)	3:22

T2 Transverse Spine	
Field Of View (mm)	130 x 91.4
Slice Thickness (mm)	3.0
Slice Gap (%)	10
Repetition Time (ms)	5200
Echo Time (ms)	101
Flip Angle (°)	150
Image Matrix	189 x 384
Bandwidth (Hz/Px)	161
Scan Time (min:sec)	5:40

EXTRAS

MPRAGE Dorsal Brain	
Field Of View (mm)	180 x 135
Slice Thickness (mm)	0.7
Slice Gap (%)	50
Repetition Time (ms)	2200
Echo Time (ms)	3.48
Flip Angle (°)	8
Image Matrix	192 x 256
Bandwidth (Hz/Px)	150
Scan Time (min:sec)	5:07

T2 FLAIR Transverse Brain	
Field Of View (mm)	140 x 85.3
Slice Thickness (mm)	3.0
Slice Gap (%)	10
Repetition Time (ms)	8500
Echo Time (ms)	122
Flip Angle (°)	150
Image Matrix	140 x 256
Bandwidth (Hz/Px)	181
Scan Time (min:sec)	4:17

CISS Transverse Spine	
Field Of View (mm)	170 x 106.3
Slice Thickness (mm)	1.0
Slice Gap (%)	20
Repetition Time (ms)	11.5
Echo Time (ms)	5.75
Flip Angle (°)	70
Image Matrix	160 x 256
Bandwidth (Hz/Px)	130
Scan Time (min:sec)	5:48

HASTE Sagittal Spine	
Field Of View (mm)	320 x 320
Slice Thickness (mm)	50.0
Slice Gap (%)	50
Repetition Time (ms)	8000
Echo Time (ms)	1200
Flip Angle (°)	150
Image Matrix	369 x 512
Bandwidth (Hz/Px)	465
Scan Time (min:sec)	0:18
