# Supplementary material for: Clinical Application of Diagnostic Imaging of Chiari-Like Malformation and Syringomyelia
Source: Front Vet Sci. 2018 Nov 28;5:280. doi: 10.3389/fvets.2018.00280 (PMC6279941; doi:10.3389/fvets.2018.00280)
Supplement: Supplementary Figure 1 — Duplicate of Figure 5 with a higher resolution. [file Image_1.pdf]

Anatomical Feature		Question	Rationale
Neural tissue	Olfactory bulbs	Size? Well-defined? Orientated rostrally?	Affected dogs are more likely to have small, ventrally orientated olfactory bulbs.
	Rostral forebrain	Rounded or flattened appearance rostrally?	Affected dogs, especially with CM pain, are more likely to have a flattened forebrain at the cribiform plate and frontal sinus.
	Whole brain conformation	If a close fitting box was drawn around the midsagittal brain, would it be a rectangle or closer to a square?	CM-pain is characterised by brachycephaly with a short skull base and a compensatory increase of height in the occipital region. As a result the “box” that would approximate the brain is closer to a “square cuboid”.
	Cerebellar shape and position	Cerebellum rounded?	Affected dogs have a cerebellum that may be flattened / indented by the supraoccipital bones and/or occipitoatlantal ligament. Alternatively/additionally it may be tilted and invaginated under the occipital lobes.
		On a mid-sagittal view, is only the cerebellum rostral to the 4th ventricle and primary fissure under the occipital lobes?	
	Cerebellar herniation	Herniation caudal to the level of the ventral edge of the supraoccipital bone?	Ubiquitous in some breeds such as the CKCS.
	Medullary position and craniospinal junction conformation.	Herniation of medulla oblongata caudal to the level of the ventral edge of the supraoccipital bone?	More severe craniocervical conformational changes increase risk of SM.
		Craniospinal junction elevated/kinked through the craniocervical junction and over the dens?	
CSF spaces	Cranial subarachnoid space	Convolutions of gyri well defined by high contrast CSF on T2W images?	Narrowing of the sulci concurrently with ventriculomegaly suggests obstruction of the CSF pathways and raised intracranial pressure.
	Lateral ventricles	Dilated? Is the corpus callosum elevated and/or thinned?	Affected dogs often have dilatation of the entire ventricular system and associated cisterns. The corpus callosum is often thinned and elevated and the tectum may be thinner and at an abnormal angle affected by dilatation of the quadrageminal cistern. By contrast the intrathalamic adhesion appears normal.
	Third ventricle and velum interpositum	Dilated? Is intrathalamic adhesion normal size?	
	Quadrageminal cistern	Dilated? Is tectum position and thickness normal?	
	Mesencephalic aqueduct	Dilated?	Suggests reduced outflow through the lateral apertures.
	Fourth ventricle	Fourth ventricle a slit (normal) or triangular (dilated) in shape?	
	Cisterna magna and spinal subarachnoid space	Reduced?	Reduced cisterna magnum and spinal subarachnoid space will affect the ability to buffer the systolic pulse and the compliance of the central nervous system.
	Fluid flow void	Fluid flow void in ventricular system?	Suggest pulsatile or turbulent flow which may be associated with progressive disease (hydrocephalous or syrinx expansion).
Bony tissue	Stop and frontal sinus	Obvious stop? Frontal sinus evident?	Affected dogs appear to have a midface insufficiency with an absent / miniscule frontal sinus with a well-defined stop and a forehead that is a layer of skin, bone then brain.
	**Cranial base** (presphenoid, basisphenoid and basioccipital)	Short appearance?	Affected dogs have a short cranial base.
	Supraoccipital bone	Midline portion present? Appear curved caudal to a vertical line drawn though the inion of the occipital crest?	In affected dogs the supraoccipital bone may be rostral to a vertical line drawn though the inion of the occipital crest. It often appears flatter. The bone is thin and the midline portion may be missing (occipital dysplasia). With extensive occipital dysplasia the cerebellum may have a more rounded shape.
	Occipital crest	Reduced?	Affected dogs often have a reduced occipital crest.
	Atlas	On a midsagittal image, when the head is in extension, where is the rostral tip of the atlas in relationship to the inion of the occipital crest?	A line drawn between the inion of the occipital crest and the rostral atlas should be angled caudally.
	Odontoid peg and axis	On a midsagittal image, when the head is in extension, what is the angulation of the dens to a basioccipital bone?	In the normal dog there is a slight curvature of craniospinal juncton over the dens. In affected dogs there is increased angulation (cervical flexure).
Ligament	Occipitoatlantal ligament / membrane	On a midline sagittal image, is this ligament horizontal (normal) or is it vertical and indenting the cerebellar vermis?	This ligamentous indentation into the cerebellum is the classic feature of CM although many confuse this with the ventral supraoccipital bone because both are hypointense. The ligament is abnormally vertical because of the close proximity of the atlas to the skull and a short supraoccipital bone.
	Atlantoaxial bands	Dorsal impingement of the subarachnoid space at C1/C2?	Noted but significance not known.
Spinal cord	Presyrinx	Is there edema (presyrinx)?	The presyrinx is a potentially reversible myelopathy that may preced development of a syrinx. It is characterised by spinal cord oedema and enlargement with no cavitation.
	Syrinx	Has the outline of spinal cord expanded?	An expanded syrinx suggests active filling and therefore more risk of progressive disease. By contrast a syrinx that is circular on transverse images and elliptical on sagittal images with little or no change to outline of the spinal cord is more likely to be asymptomatic.
		Symmetrical on sagittal images ?	
		On transverse images, what is maximum diameter of the syrinx in the cervical, thoracic and thoracolumbar regions?	Symptomatic disease is more likely with a syrinx diameter of 4mm or more (CKCS).
		Does the syrinx location fit with the neurolocalisation (region of spinal cord and within spinal cord)?	Phantom scratching is associated with extension to the superficial dorsal horn in the C3-C6 spinal cord segments (corresponding to C2-C5 vertebrae). Cervicotorticollis / scoliosis are associated with extension of the syrinx into the superficial dorsal horn ipsilateral to the phantom scratching side and /or contralateral to the head tilt. Paresis and proprioceptive deficits are associated with large syringes in an appropriate region of the spinal cord.
		Is there dorsal horn involvement?	
		Is there fluid flow void?	Suggest pulsatile or turbulent flow (slosh effect) and increase risk of syrinx expansion.

Front. Vet. Sci. doi: 10.3389/fvets.2018.00280
Clare Rusbridge ©2018
